# The SIK1/CRTC2/CREB1 and TWIST1/PI3K/Akt/GSK3β signaling pathways mediated by microRNA-25-3p are altered in the schizophrenic rat brain

**DOI:** 10.3389/fncel.2023.1087335

**Published:** 2023-01-20

**Authors:** Bo Pan, Xiaoli Zhu, Bing Han, Jianjun Weng, Yuting Wang, Yanqing Liu

**Affiliations:** ^1^The Key Laboratory of Syndrome Differentiation and Treatment of Gastric Cancer of the State Administration of Traditional Chinese Medicine, Yangzhou University Medical College, Yangzhou, China; ^2^Institute of Translational Medicine, Yangzhou University Medical College, Yangzhou, China

**Keywords:** schizophrenia, microRNA-25-3p, SIK1/CRTC2/CREB1 signaling pathway, TWIST1/PI3K/Akt/GSK3β signaling pathway, risperidone

## Abstract

Schizophrenia is a group of severe mental disorders. MiR-25-3p was shown to be involved in various neuropsychiatric diseases and can regulate SIK1 and TWIST1. The CRTC2/CREB1 and PI3K/Akt/GSK3β signaling pathways are downstream pathways of SIK1 and TWIST1, respectively. This study investigated whether miR-25-3p-mediated SIK1/CRTC2/CREB1 and TWIST1/PI3K/Akt/GSK3β signaling pathways are present in an animal model relevant to schizophrenia. A schizophrenic rat model was established by using sub-chronic MK-801 administration. An RNA-seq test was performed to examine the differentially expressed genes (DEGs) in the rat prefrontal cortex (PFC). The mRNA levels of miR-25-3p, SIK1, and TWIST in the PFC and caudate putamen (CPu) were assessed by qRT-PCR. Phosphorylation of the SIK1/CRTC2/CREB1 and TWIST1/PI3K/Akt/GSK3β pathways in the two brain regions was examined by Western blots. The RNA-seq data revealed down-regulated miR-25-3p expression and up-regulated SIK1 and TWIST1 mRNA expression induced by MK-801. Additionally, SIK1 and TWIST1 were shown to be possible downstream responders of miR-25-3p in previous studies. qRT-PCR confirmed the changes of miR-25-3p, SIK1, and TWIST1 induced by MK-801 in both brain regions, which, however, was reversed by risperidone. Furthermore, the phosphorylation of the SIK1/CRTC2/CREB1 pathway was repressed by MK-801, whereas the phosphorylation of the TWIST1/PI3K/Akt/GSK3β pathway was increased by MK-801 in either of the two brain regions. Moreover, the altered phosphorylation of these two signaling pathways induced by MK-801 can be restored by risperidone. In conclusion, this study suggests that altered SIK1/CRTC2/CREB1 and TWIST1/PI3K/Akt/GSK3β signaling pathways mediated by miR-25-3p is very likely to be associated with schizophrenia, revealing potential targets for the treatment and clinical diagnosis of schizophrenia.

## 1. Introduction

Schizophrenia is a group of common, serious mental disorders, affecting approximate 1% general population and largely increasing patients’ individual and social burdens (McCutcheon et al., 2020). However, the exact pathophysiological mechanism of schizophrenia remained to be elucidated. To date, limited cellular signaling pathways have been reported to be related to schizophrenia. Thus, discovering and investigating new cellular signaling pathways will be of vital importance for developing novel treatment and drugs of schizophrenia.

MicroRNAs are short (20–24 nt) non-coding RNAs that influence the stability and translation of mRNAs and regulate post-transcriptional gene expression, and the dysfunction of microRNAs has been reported to be associated with a wide range of diseases. Previous studies showed that miR-25 is involved in the pathogenesis of various diseases, such as acute myocardial infarction, left ventricular hypertrophy, heart failure, diabetes mellitus, diabetic nephropathy, tubulointerstitial nephropathy, and asthma bronchiale (Sárközy et al., 2018). Moreover, miR-25 was found to play a critical role in the pathogenesis of a range of neuropsychiatric diseases, including epilepsy (Li et al., 2020), Parkinson’s disease (Chen et al., 2020), cerebral ischemia (Guo et al., 2014; Zhang et al., 2016; Zhou and Qiao, 2022), multiple sclerosis (Nuzziello et al., 2018), panic disorder (Min et al., 2019), alexithymia (Van der Auwera et al., 2022), and tic disorders (Wang et al., 2022). It was also reported that sarco(endo)plasmic reticulum Ca(2+) ATPase (SERCA2), a downstream target of miR-25, was elevated in the brains of patients with schizophrenia (Earls et al., 2012), suggesting a potential connection between miR-25 and schizophrenia. In the present study, such connection was further assessed.

MiR-25 is the regulator of many signaling pathways. A previous study indicates that salt-inducible kinase 1 (SIK1) can be directly bound and regulated by miR-25-3p in hepatocellular carcinoma (Fu et al., 2022). The SIK family, consisting of three members–SIK1, SIK2, and SIK3, closely resembles the AMP-activated protein kinase (AMPK) and other AMPK-related kinases (Darling and Cohen, 2021). They are required to be phosphorylated by liver kinase B1 (LKB1) to be catalytically active (Lizcano et al., 2004) and can regulate macrophage function in the immune system, cytokines secretion from mast cells, bone development and remodeling, tumorigenesis, circadian rhythms and sleep, melanin production in the skin, and metabolism (Darling and Cohen, 2021). In particular, SIK1 and SIK2 were found to be associated with neuropsychiatric disorders, such as epilepsy and depression, and inhibition of SIKs was shown to be an effective measure to treat these neuropsychiatric disorders (Hansen et al., 2015; Pröschel et al., 2017; Liu et al., 2020). However, whether normalizing SIKs is a therapeutic measure for schizophrenia has not been reported.

The phosphorylation and activation of SIKs requires LKB1 and phosphorylated SIKs repress the transcription factor cAMP-response element binding protein (CREB) by phosphorylating CREB-regulated transcriptional co-activators (CRTCs) (Darling and Cohen, 2021). Additionally, it has been well documented that a reduced CREB1 activity is highly connected with the pathophysiology of schizophrenia (Wang et al., 2018). Since it was previously reported that CRTC1 is expressed almost exclusively in the hypothalamus and CRTC3 is expressed in adipose tissue (Altarejos and Montminy, 2011), the SIK1/CRTC2/CREB1 signaling pathway was selected and its involvement in the pathophysiology of schizophrenia was investigated in this study.

A previous study revealed that Twist Family bHLH (basic helix-loop-helix) Transcription Factor 1 (TWIST1) is a direct downstream responder of miR-25-3p (Yu et al., 2021). TWIST1 is a bHLH domain-containing transcription factor, playing an essential role in morphogenesis and organogenesis, such as mesodermal formation, neurogenesis, myogenesis, and neural crest cell migration and differentiation (Qin et al., 2012). TWIST1 expression was found in several cancers and promoted cancer cell invasion and metastasis (Qin et al., 2012). In addition, in the central nervous system (CNS), TWIST1 was shown to be expressed in embryonic and fetal human brain neurons (Elias et al., 2005), indicating that its dysfunction might be associated with neuropsychiatric diseases. For example, a previous study reported that the expression of TWIST1 was positively correlated with depressive behaviors in humans and mice; more specifically, elevated TWIST1 expression induced by chronic stress was found in the mice medial prefrontal cortex, hippocampus, striatum, etc. (He et al., 2021). Due to the close relationship between TWIST1 and neurodevelopment, it is worth investigating whether TWIST1 is involved in the pathogenesis of schizophrenia which is a neurodevelopmental mental disorder.

Previous studies indicated that TWIST1 expression can induce Akt signaling through either causing Akt phosphorylation (Li and Zhou, 2011; Roberts et al., 2016) or altering its protein and mRNA expression (Cheng et al., 2007). Additionally, LY294002, a PI3K inhibitor, was able to inhibit the Akt activation induced by TWIST1 (Roberts et al., 2016). Furthermore, altered Akt/GSK3β signaling was widely reported to be associated with schizophrenia (Emamian, 2012); and, it was pointed out that GSK3β can be phosphorylated at Ser9 by TWIST1 through the activation of the PI3K/Akt (Ser473) signaling (Li and Zhou, 2011). Therefore, the present study further explored whether the TWIST1/Akt/PI3K/GSK3β signaling pathway is abnormally modulated in schizophrenia.

Overall, this study investigated the role of miR-25-3p in schizophrenia by using bioinformatic methods and a sub-chronic pharmacological rat model of schizophrenia. In addition, two possible downstream pathways of miR-25-3p, including the SIK1/CRTC2/CREB1 and TWIST1/PI3K/Akt/GSK3β signaling pathways, were examined with the schizophrenic rat model to explore their potential associations with schizophrenia.

## 2. Materials and methods

### 2.1. Animals

Male Sprague-Dawley (SD) rats (aging 21 days and weighing 60 ± 5 g) were purchased from the Chang Cavens Laboratory Animals Co., Ltd. (Changzhou, Jiangsu, China). Each rat was placed in a single cage with food and water *ad libitum*. After 7-day habituation in a controlled environment (22 ± 1°C; light cycle from 07:00 a.m. to 07:00 p.m.), the rats were randomly assigned into three groups (*n* = 6 per group). The drug administration was performed as described previously (Pan et al., 2022a,b, 2023). The experimental procedures of the present study were approved by the Animal Ethics Committee of Yangzhou University Medical College (Ethics No.: YXYLL-2020-53).

### 2.2. Drug administration regimen

MK-801 is a non-competitive N-methyl-D-aspartic acid receptor (NMDAR) antagonist and the NMDAR hypofunction models employing MK-801 are commonly-used schizophrenic animal models (Ang et al., 2021). The drug administration initiated after 1-week habituation. Briefly, the MK-801 and risperidone groups received daily intraperitoneal injections of MK-801 (#M107, Sigma-Aldrich, St. Louis, MO, USA) at a dose of 0.2 mg/kg between 10:00 a.m. and 10:30 a.m. for 2 weeks, and the control group were intraperitoneally injected saline (0.9%) for comparison. Our previous study showed that this MK-801 administration method successfully induced schizophrenia-like symptoms in SD rats (Pan et al., 2022b). After the 2-week MK-801 administration, the risperidone group were orally administrated with risperidone (0.3 mg/kg, Xian Janssen Pharmaceutical Ltd., Xi’an, Shaanxi, China) three times a day (07:00 a.m., 3:00 p.m., and 11:00 p.m.) for 2 weeks. As previously described (Pan et al., 2022a,b, 2023), risperidone tablets were first ground, and then the drug powder was mixed with cookie dough pellets and orally delivered to animals. The other two groups were administrated with equivalent cookie dough pellets without risperidone. Two hours after the last oral administration, all rats were sacrificed and the whole brains were removed and then quickly frozen and stored under −80°C for future use.

### 2.3. Brain sample dissection

Prefrontal cortex (PFC) and caudate putamen (CPu) are the brain regions that were found to be closely related to schizophrenia (Buchsbaum et al., 2003; Manoach, 2003). In this study, the PFC and CPu samples were collected using frozen microdissection, as described previously (Pan et al., 2018, 2022a,b; Pan and Deng, 2019). Briefly, PFC slides through the forebrain (Bregma 4.68 to 2.76 mm) and CPu slides through the striatum (Bregma 2.28 to 0.96 mm) were dissected using a cryostat (#CM1860, Leica Biosystems, Nussloch, Germany) (Paxinos and Watson, 2007). Then, tissue punches (Φ1 mm for PFC and Φ3 mm for CPu) were used to collect tissue samples bilaterally from the PFC and CPu slides, respectively.

### 2.4. RNA sequencing

Two groups of male SD rats (*n* = 5 per group) were received either MK-801 (0.2 mg/kg) or saline (0.9%) intraperitoneal injections for 2 weeks, as described in the previous Section “2.3 Brain sample dissection.” PFC tissue samples were collected freshly when sacrificing the rats and then quickly frozen and stored under −80°C for RNA sequencing.

The RNA-seq test was performed by Novogene (Beijing, China). Briefly, the RNA-seq library for sequencing was prepared by Novogene following the standard Illumina protocols. Reads were aligned to the genome using HISAT2 (Version 2.2.1) and SAMtools (Version 1.15.1) with referencing the gene annotation file from Ensembl (Release 105) (Li et al., 2009; Kim et al., 2019). FeatureCounts (Subread package Version 2.0.1) was employed to count the reads and then to create the expression matrix (Liao et al., 2014). Differentially-expressed genes (DEGs) were determined by using DESeq2 (Version 1.36.0) with *P*-value < 0.05 and | logFC| ≥ 1 as the selection criteria. Functional enrichment for Gene Ontology (GO) were performed and visualized using online tools in http://www.bioinformatics.com.cn. Heatmap, volcano plot, and Venn diagram in the present study were created using pheatmap (Version 1.0.12), ggplot2 (Version 3.3.6), and VennDiagram (Version 1.7.3) in R, respectively.

### 2.5. RNA extraction and quantitative real-time PCR

The methods of RNA extraction and quantitative real-time PCR (qRT-PCR) were described previously (Pan et al., 2022a,b). Briefly, total RNA and microRNA was extracted from rat brain tissues using RNeasy Mini Kit (#74106, Qiagen, Germantown, MD, USA) and PureLink miRNA Isolation Kit (#K157001, ThermoFisher Scientific), respectively, following the manufacturers’ instructions. Then, the extracted total RNA and microRNA was reversely transcribed using PrimeScript™ RT Master Mix (#RR036A, TaKaRa, Shiga, Japan) and Mir-XTM miRNA First-Strand Synthesis Kit (#638315, TaKaRa), respectively. A LightCycler^®^ 96 System (Roche Molecular Systems, Mannheim, Germany) with TB Green^®^ Premix EX Taq™ II (#RR820A, TaKaRa) was employed to carry out qRT-PCR experiments. U6 was served as an internal control. The parameters of qRT-PCR are as follows: preincubation 95°C for 30 s, followed by 40 cycles of 95°C for 15 s and 60°C for 60 s.

Sequences of the primers are listed as follows:

*miRNA-25-3p*, forward primer: 5′-CATTGCACTTGTCTCG GTCTGA-3′, reverse primer: 5′-CCGAGGTATAGATGATGTAC TA-3′

*SIK1*, forward primer: 5′- GCATACACTGGCTGAAGTTTCCAC-3′, reverse primer: 5′-CAGGACCTTCGCTTGCAGA-3′

*TWIST1*, forward primer: 5′- TGCAGACACAGCGGGTCAT-3′, reverse primer: 5′-GGTCTGAATCTTGCTCAGCTTGT-3′

*U6* (internal control), forward Primer 5′-GGAACGATACAGAG AAGATTAGC-3′, reverse primer: 5′-TGGAACGCTTCACGAA TTTGCG-3′

### 2.6. Western blots

Western blots were performed as previously described (Pan et al., 2018, 2022a; Pan and Deng, 2019). Briefly, the brain samples were homogenized in a NP-40 homogenizing buffer (#P0013F, Beyotime) containing Protease Inhibitor Cocktail (#P8340, Sigma-Aldrich, St. Louis, MO, USA). Loading samples containing 15 μg of total protein were loaded into a 12% SDS-PAGE gel, followed by transferring proteins from the gel to a polyvinylidene difluoride (PVDF) membrane. The PVDF membrane with proteins on it was blocked by 5% skim milk for 2 h at room temperature. Then, the prepared membrane was incubated with a primary antibody overnight at 4°C and a secondary antibody for 2 h at room temperature on the next day. The immunoreactive signals were obtained by the ChemiDoc XRS + System (Bio-Rad, Hercules, CA, USA) and quantified by ImageLab™ Software (Bio-Rad, Version 6.1). The data were obtained by normalizing with their corresponding β-actin or GAPDH levels and then transferring by taking the value of the control group as 100%. The images of the full membranes of the signal bands demonstrated in this study are shown in the Supplementary Figures 1–4.

The primary antibodies used in this study are listed as follows: anti-SIK1 (1:1000; #K009180P, Solarbio, Beijing, China), anti-Phospho-SIK1 (Thr182) (1:1000; #abs140018, Absin, Shanghai, China), anti-TWIST1 (1:1000; #K009993P, Solarbio), anti-Akt (pan) (C67E7) (1:2000; #4691, Cell Signaling, Danvers, MA, USA), anti-Phospho-Akt (Ser473) (1:2000; #4060, Cell Signaling), anti-PI3 Kinase p85 (1:2000; #4292, Cell Signaling), anti-Phospho-PI3-kinase p85-alpha (Tyr607) (1:1000; #abs130868, Absin), anti-GSK3β (1:1000; #abs158138, Absin), anti-Phospho-GSK3β (Ser9) (1:1000, #abs130628, Absin), anti-LKB1 (1:1000, #abs147256, Absin), anti-CREB1 (1:2000; #12208-1-AP, Proteintech, Rosemont, IL, USA), anti-Phospho-CREB1 (Ser133) (1:1000; #abs154911, Absin), anti-CRTC2 (1:1000; #abs131124, Absin), and anti-Phospho-CRTC2 (Ser171) (1:1000; #abs140157, Absin). Mouse anti-actin polyclonal antibody (1:10000; #3700, Cell Signaling) and mouse GAPDH monoclonal antibody (1:50000; #60004-1-lg, Proteintech) were used to determine β-actin and GAPDH levels, respectively. The secondary antibodies used in this study include HRP-linked anti-mouse IgG antibody (1:2000; #7076, Cell Signaling) and HRP-linked anti-rabbit IgG antibody (1:2000; #7074, Cell Signaling).

### 2.7. Statistics

The RNA-seq data were analyzed as described in the previous Section “2.6 Western blots.” The data of the qRT-PCR and Western blots experiments were analyzed and visualized using Prism GraphPad (Version 9.4.1) (GraphPad Software, San Diego, CA, USA). Data normal distribution was determined using histograms and Kolmogorov–Smirnov *Z*-tests. One-way analysis of variance (ANOVA) analysis was performed if the data were normally distributed, followed by performing *post-hoc* Dunnett’s *t-*tests to compare each group with their corresponding model (MK-801) groups. Statistical significance was considered when *P-*value was less than 0.05. All experiments were performed in triplicate to ensure consistency.

## 3. Results

### 3.1. MiR-25-3p was down-regulated in the schizophrenic rat brain, accompanied with up-regulated mRNA expression of SIK1 and TWIST1

**FIGURE 1:**
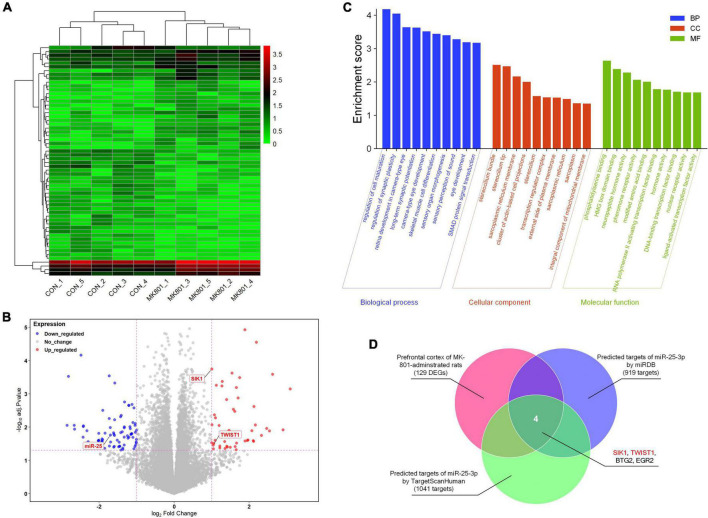
RNA-seq analysis of the differentially-expressed genes in the prefrontal cortex. **(A)** Heatmap of the differentially-expressed genes. **(B)** Volcano plot of the differentially-expressed genes. **(C)** Gene Ontology (GO) enrichment analysis of the differentially-expressed genes. **(D)** Venn diagram of the genes that might be associated with schizophrenia and also are potential targets of miR-25-3p.

The RNA-seq analysis of the rat PFC samples (GSE220510) revealed 129 DEGs, including 50 down-regulated and 79 up-regulated DEGs (Figures 1A, B). In addition, GO enrichment analysis showed that enriched GO terms mainly include regulation of cell maturation, regulation of synaptic plasticity, long-term synaptic potentiation, transcription regulator complex, phosphatidylserine binding, neuropeptide hormone activity, RNA polymerase II activating transcription factor binding, DNA-binding transcription factor binding, and so forth (Figure 1C and Supplementary Table 1). By searching with the online database TargetScanHuman (Release 8.0) and miRDB (Version 6.0), 1,041 and 919 potential targets of has-miR-25-3p were found, respectively. The Venn diagram showed that there are four genes (*SIK1, TWIST1, BTG2, and EGR2*) that were altered in the schizophrenic model rat PFC and also are potential targets of miR-25 (Figure 1D), among which SIK1 and TWIST1 are of our particular interests, since there is no previous study reporting their associations with schizophrenia.

**FIGURE 2:**
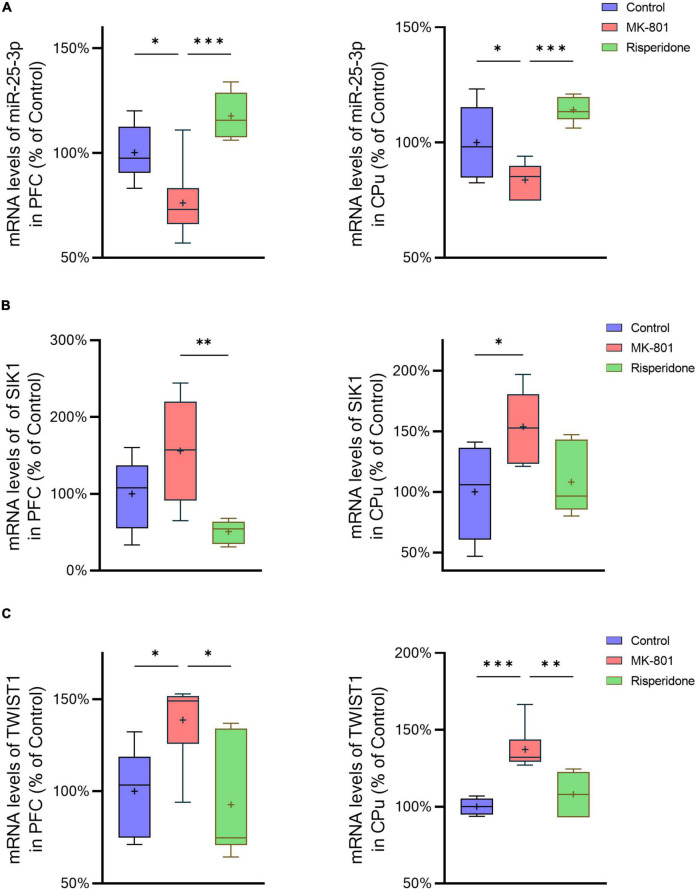
Altered mRNA expression of miR-25-3p, SIK1, and TWIST1 induced by the repeated MK-801 administration in the prefrontal cortex and caudate putamen. **(A)** The mRNA expression of miR-25-3p was decreased in the model rat prefrontal cortex (PFC) and caudate putamen (CPu), which was restored by risperidone. **(B)** The SIK1 mRNA expression were up-regulated in the model rat PFC and CPu; risperidone also reversed these alterations. **(C)** The TWIST1 mRNA expression was inhibited in the model rat PFC and CPu, which can be reversed to near normal levels by risperidone. *P < 0.05, ^**^P < 0.01, ^***^P < 0.001, + mean value.

The mRNA expression of miR-25-3p, SIK1, and TWIST1 in the PFC and caudate putamen (CPu) was detected by qRT-PCR. The results showed that the repeated MK-801 administration significantly reduced the mRNA expression of miR-25-3p in both of the PFC (ANOVA: *F*_2,15_ = 12.4, *P* < 0.001; *post-hoc*: −24.0%, *P* < 0.05 vs. MK-801 group) and CPu (ANOVA: *F*_2,15_ = 12.4, *P* < 0.001; *post-hoc*: –16.3%, *P* < 0.05 vs. MK-801 group) (Figure 2A). However, risperidone eliminated these inhibitory effects of MK-801 (both *P* < 0.05 vs. MK-801 group) (Figure 2A). Additionally, the MK-801 administration elevated the mRNA expression of SIK1 in the PFC, but not achieving significance (ANOVA: *F*_2,15_ = 7.14, *P* < 0.01; *post-hoc*: +55.7%, *P* = 0.11 vs. MK-801 group); on the other hand, the inhibitory effect of MK-801 on SIK1 in the CPu was statistically significant (ANOVA: *F*_2,15_ = 4.804, *P* < 0.05; *post-hoc*: +53.67%, *P* < 0.05 vs. MK-801 group) (Figure 2B). Furthermore, the mRNA levels of TWIST1 of the MK-801 group were significantly higher than those of the control group in both of the PFC (ANOVA: *F*_2,15_ = 8.34, *P* < 0.01; *post-hoc*: +38.7%, *P* < 0.01 vs. MK-801 group) and CPu (ANOVA: *F*_2,15_ = 15.82, *P* < 0.001; *post-hoc*: + 37.19%, *P* < 0.001 vs. MK-801 group) (Figure 2C). It needs to be mentioned that the altered mRNA levels of both SIK1 and TWIST1 in the two brain regions could be restored by risperidone to near normal levels (in the PFC: *P* < 0.05 vs. MK-801 group; in the CPu: *P* < 0.01 vs. MK-801 group) (Figures 2B, C).

### 3.1. The SIK1/CRTC2/CREB1 signaling is repressed in the schizophrenic rat brain

**FIGURE 3:**
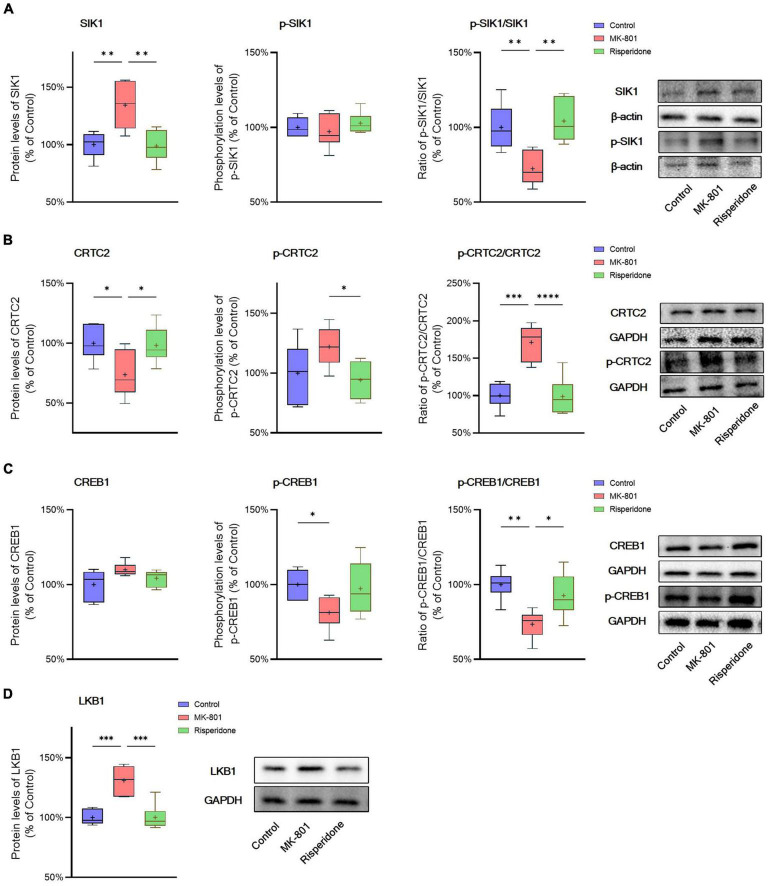
Altered SIK1/CRTC2/CREB1 signaling induced by the repeated MK-801 administration in the prefrontal cortex. **(A)** The SIK1 phosphorylation was repressed by the MK-801 administration. **(B)** The CRTC2 phosphorylation in the model rat prefrontal cortex (PFC) was elevated, and reduced to near normal levels by risperidone. **(C)** The CREB1 phosphorylation CREB1 in the model rat PFC was down-regulated, but restored by risperidone. **(D)** The LKB1 protein expression was up-regulated by the MK-801 administration, but reversely regulated by risperidone. *P < 0.05, ^**^P < 0.01, ^***^P < 0.001, ^****^P < 0.0001, + mean value.

**FIGURE 4:**
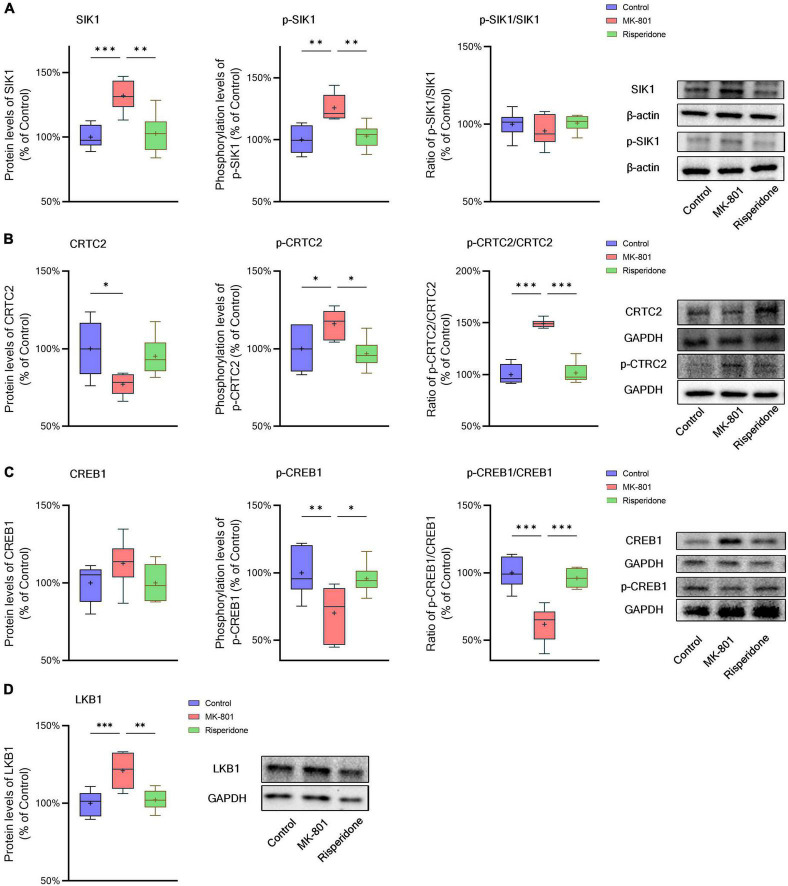
Altered SIK1/CRTC2/CREB1 signaling induced by the repeated MK-801 administration in the caudate putamen. **(A)** The protein expression and phosphorylation levels of SIK1 were altered by MK-801, but the ratio of p-SIK1/SIK1 did not change. **(B)** The phosphorylation of CRTC2 in the model rat caudate putamen (CPu) was up-regulated by MK-801, but reversed by risperidone. **(C)** The phosphorylation of CREB1 in the model rat CPu was inhibited, which was restored by risperidone. **(D)** The protein expression of LKB1 was increased by MK-801, which was also eliminated by risperidone. *P < 0.05, ^**^P < 0.01, ^***^P < 0.001, + mean value.

The total protein and phosphorylation levels of SIK1, CRTC2, and CREB1 as well as the LKB1 total protein expression in the rat PFC and CPu were examined by Western blots. Compared with the control group, the MK-801 administration significantly elevated the total protein expression of SIK1 in the PFC (ANOVA: *F*_2,15_ = 10.2, *P* < 0.01; *post-hoc*: +34.4%, *P* < 0.01 vs. MK-801 group); although the phosphorylation levels of SIK1 did not differ significantly among the three groups, the ratio of p-SIK1/SIK1 was decreased in the MK-801 group (ANOVA: *F*_2,15_ = 9.755, *P* < 0.01; *post-hoc*: −27.59%, *P* < 0.01 vs. MK-801 group) (Figure 3A). Similarly, risperidone exerted inverse effects which eliminated the influences of MK-801 on the SIK1 phosphorylation in the PFC (all *P* > 0.05 vs. MK-801 group) (Figure 3A). In the CPu, MK-801 significantly increased both of the total protein expression (ANOVA: *F*_2,15_ = 12.3, *P* < 0.001; *post-hoc*: +32.0%, *P* < 0.001 vs. MK-801 group) and phosphorylation levels of SIK1 (ANOVA: *F*_2,15_ = 10.3, *P* < 0.01; *post-hoc*: +25.7%, *P* < 0.01 vs. MK-801 group); however, no significant difference in the ratio of p-SIK1/SIK1 was found in this brain area (ANOVA: *F*_2,15_ = 0.696, *P* > 0.05) (Figure 4A).

Furthermore, in the PFC, the repeated MK-801 administration significantly down-regulated the CRTC2 protein expression (ANOVA: *F*_2,15_ = 4.752, *P* < 0.05; *post-hoc*: −26.21%, *P* < 0.05 vs. MK-801 group). Although the phosphorylation levels of CRTC2 were not significantly altered by MK-801 (ANOVA: *F*_2,15_ = 3.402, *P* > 0.05), the ratio of p-CRTC2/CRTC2 of the model group was still higher than that of the control group (ANOVA: *F*_2,15_ = 21.0, *P* < 0.0001; *post-hoc*: +71.1%, *P* < 0.001 vs. MK-801 group), but reversed to near normal levels by risperidone (*P* < 0.0001 vs. MK-801 group) (Figure 3B). In the CPu, the CRTC2 protein expression was significantly repressed by MK-801 (ANOVA: *F*_2,15_ = 5.09, *P* < 0.05; *post-hoc*: −22.9%, *P* < 0.05 vs. MK-801 group); however, the phosphorylation levels of CRTC2 (ANOVA: *F*_2,15_ = 5.01, *P* < 0.05; *post-hoc*: +16.0%, *P* < 0.05 vs. MK-801 group) and the ratio of p-CRTC2/CRTC2 (ANOVA: *F*_2,15_ = 67.4, *P* < 0.001; *post-hoc*: +49.3%, *P* < 0.001 vs. MK-801 group) were significantly elevated in the model rat CPu; similarly, these MK-801-induced changes in the CRTC2 phosphorylation could be restored by risperidone (p-CRTC2: *P* < 0.05 vs. MK-801 group; ratio of p-CRTC2/CRTC2: *P* < 0.001 vs. MK-801 group) (Figure 4B).

The total protein expression of CREB1 was not affected by MK-801 in both brain regions. However, the phosphorylation levels of CREB1 were significantly down-regulated in both of the PFC (ANOVA: *F*_2,15_ = 3.56, *P* < 0.05; *post-hoc*: −18.8%, *P* < 0.01 vs. MK-801 group) (Figure 3B) and CPu (ANOVA: *F*_2,15_ = 5.46, *P* < 0.05; *post-hoc*: −29.8%, *P* < 0.01 vs. MK-801 group) of the model group (Figure 4B). Similarly, the ratio of p-CREB1/CREB1 of the MK-801 group in these two brain areas was also significantly lower (PFC: ANOVA: *F*_2,15_ = 8.58, *P* < 0.01; *post-hoc*: −26.5%, *P* < 0.01 vs. MK-801 group. CPu: ANOVA: *F*_2,15_ = 22.3, *P* < 0.001; *post-hoc*: −38.2%, *P* < 0.001 vs. MK-801 group) (Figures 3C, 4C). Moreover, risperidone was able to reverse the down-regulated phosphorylation of CREB1 induced by the repeated MK-801 administration in both brain regions (all *P* < 0.05 or 0.01 vs. MK-801 group) (Figures 3B, C, 4B, C).

It was reported that SIK1 phosphorylation is dependent on LKB1 (Lizcano et al., 2004). Thus, the protein expression of LKB1 was examined in these two brain regions as well. In the PFC, the MK-801 administration significantly elevated the LKB1 expression (ANOVA: *F*_2,15_ = 19.14, *P* < 0.0001; *post-hoc*: + 30.89%, *P* < 0.001 vs. MK-801 group), which, however, was reversed by risperidone (*P* < 0.001 vs. MK-801 group) (Figure 3D); in the CPu, the LKB1 levels were also significantly increased in the model group (ANOVA: *F*_2,15_ = 10.4, *P* < 0.01; *post-hoc*: +21.0%, *P* < 0.001 vs. MK-801 group) and decreased to near normal levels by risperidone (*P* < 0.01 vs. MK-801 group) (Figure 4D).

### 3.3. TWIST1/PI3K/Akt/GSK3β signaling is inhibited in the schizophrenic rat brain

**FIGURE 5:**
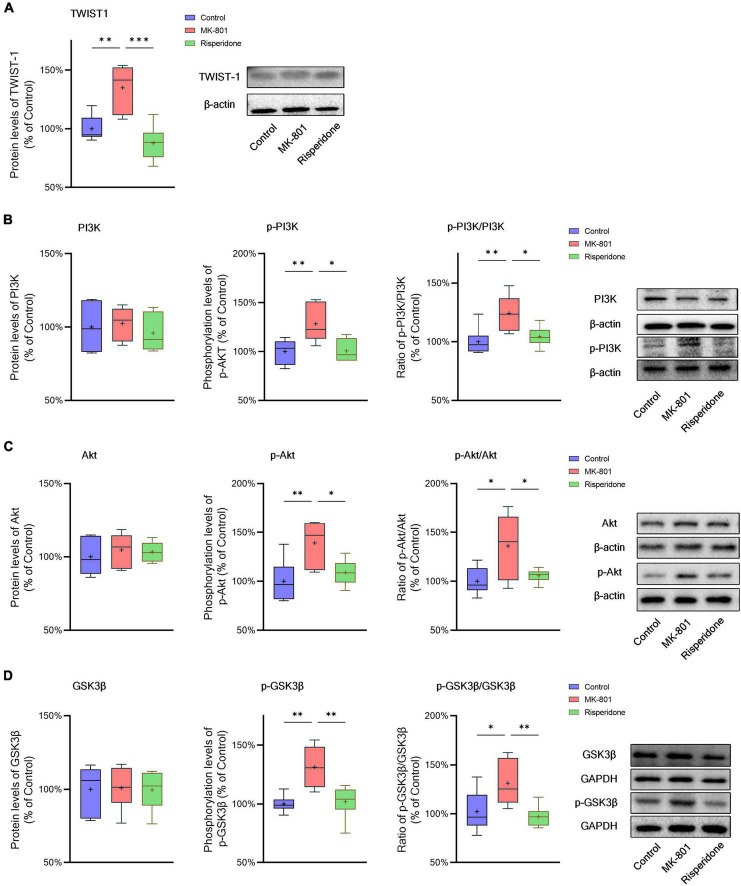
Changed TWIST1/PI3K/Akt/GSK3β signaling induced by the repeated MK-801 administration in the prefrontal cortex. **(A)** The protein expression of TWIST1 in the model rat prefrontal cortex (PFC) was inhibited, but returned to normal levels after administrating with risperidone. **(B)** The MK-801 administration reduced PI3K phosphorylation in the model rat PFC, which can also be reversed by risperidone. **(C)** The Akt activation in the model rat PFC was decreased, but up-regulated to near normal levels by risperidone. **(D)** The repeated MK-801 administration inhibited the GSK3β phosphorylation in the model rat PFC, but restored by risperidone. *P < 0.05, ^**^P < 0.01, ^***^P < 0.001, + mean value.

**FIGURE 6:**
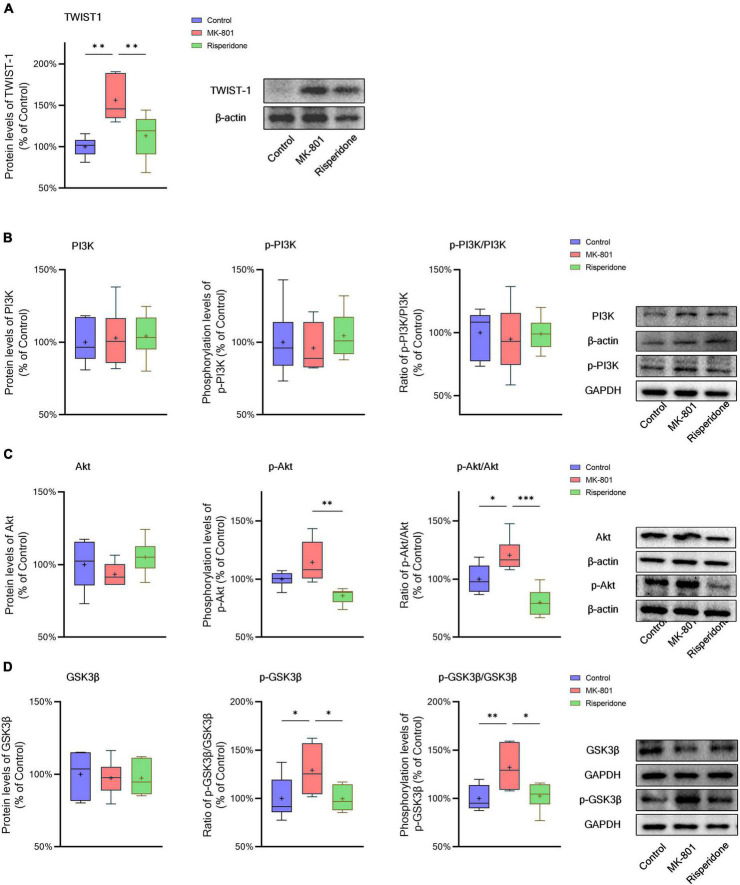
Changed TWIST1/PI3K/Akt/GSK3β signaling induced by the repeated MK-801 administration in the caudate putamen. **(A)** The protein levels of TWIST1 in the model rat caudate putamen (CPu) was reduced, but restored by risperidone. **(B)** The PI3K phosphorylation in the model rat CPu was not altered in the model rat CPu. **(C)** The phosphorylation of Akt in the model rat CPu was inhibited, but reversed by risperidone. **(D)** The GSK3β phosphorylation was repressed by the MK-801 administration, but reversely affected by risperidone. *P < 0.05, ^**^P < 0.01, ^***^P < 0.001, + mean value.

The protein expression of TWIST1 and phosphorylation of PI3K, Akt, and GSK3β in the rat PFC and CPu were assessed by Western blots. In comparison with the control group, the protein levels of TWIST1 were significantly increased in both of the PFC (ANOVA: *F*_2,15_ = 14.7, *P* < 0.01; *post-hoc*: +35.0%, *P* < 0.01 vs. MK-801 group) (Figure 5A) and CPu (ANOVA: *F*_2,15_ = 9.94, *P* < 0.01; *post-hoc*: +56.2%, *P* < 0.01 vs. MK-801 group) (Figure 6A), which, however, were reversed by risperidone (both *P* < 0.001 or 0.01 vs. MK-801 group) (Figures 5A, 6A).

Additionally, without significantly changing the protein levels of PI3K, the MK-801 administration increased the PI3K phosphorylation levels (ANOVA: *F*_2,15_ = 7.09, *P* < 0.01; *post-hoc*: +28.3%, *P* < 0.01 vs. MK-801 group) and the ratio of p-PI3K/PI3K (ANOVA: *F*_2,15_ = 6.29, *P* < 0.05; *post-hoc*: +24.2%, *P* < 0.01 vs. MK-801 group) in the PFC (Figure 5B), both of which were restored by risperidone (both *P* < 0.05 vs. MK-801 group); in the CPu, neither the PI3K protein expression nor its phosphorylation levels were significantly affected by the MK-801 administration (all *P* > 0.05 vs. MK-801 group) (Figure 6B).

It was previously reported that repeated administration of MK-801 can induce the phosphorylation of Akt/GSK3β signaling in the rat PFC (Seo et al., 2007; Park et al., 2014; Jeon et al., 2017). In the present study, although the Akt protein expression did not differ among the three groups, MK-801 increased the phosphorylation levels of Akt (ANOVA: *F*_2,15_ = 6.48, *P* < 0.01; *post-hoc*: +39.1%, *P* < 0.01 vs. MK-801 group) as well as the ratio of p-Akt/Akt (ANOVA: *F*_2,15_ = 5.21, *P* < 0.05; *post-hoc*: +36.0%, *P* < 0.05 vs. MK-801 group) in the PFC (Figure 5C); in the CPu, without significantly altering the Akt phosphorylation levels (ANOVA: *F*_2,15_ = 9.47, *P* < 0.01; *post-hoc*: +14.5%, *P* = 0.08 vs. MK-801 group), the ratio of p-Akt/Akt of the MK-801 group was significantly increased (ANOVA: *F*_2,15_ = 15.2, *P* < 0.001; *post-hoc*: +20.6%, *P* < 0.05 vs. MK-801 group) (Figure 6C).

Furthermore, the alterations in the GSK3β phosphorylation of the two brain regions were as similar as those in Akt. Specifically, although the protein expression of GSK3β in the PFC was not significantly different from that of the model group, the phosphorylation levels of GSK3β (ANOVA: *F*_2,15_ = 10.00, *P* < 0.01; *post-hoc*: +31.45%, *P* < 0.01 vs. MK-801 group) and the ratio of p-GSK3β/GSK3β was both significantly increased by MK-801 (ANOVA: *F*_2,15_ = 5.60, *P* < 0.05; *post-hoc*: +26.0%, *P* < 0.05 vs. MK-801 group) (Figure 5D); in the CPu, the MK-801 administration significantly elevated both of the GSK3β phosphorylation levels (ANOVA: *F*_2,15_ = 6.64, *P* < 0.01; *post-hoc*: +32.1%, *P* < 0.01 vs. MK-801 group) and the ratio of p-GSK3β/GSK3β (ANOVA: *F*_2,15_ = 4.06, *P* < 0.05; *post-hoc*: +29.2%, *P* < 0.05 vs. MK-801 group), but not the total protein expression of GSK3β (Figure 6D). It should be also noted that risperidone was able to eliminate the inhibitory effects of MK-801 on the Akt/GSK3β signaling in both brain regions (all *P* < 0.05, 0.01, or 0.001 vs. MK-801 group) (Figures 5C, D, 6C, D).

## 4. Discussion

The present study found that the miR-25-3p-mediated SIK1/CRTC2/CREB1 and TWIST1/PI3K/Akt/GSK3β signaling pathways were altered in the brains of schizophrenic model rats. To the best of our knowledge, this is the first study that reveals a possible association between miR-25-3p and schizophrenia and potential involvements of the SIK1/CRTC2/CREB1 and TWIST1/PI3K/Akt/GSK3β signaling pathways in schizophrenia. Furthermore, the current study also showed that the altered SIK1/CRTC2/CREB1 and TWIST1/PI3K/Akt/GSK3β signaling pathways in the schizophrenic model rat brains could be restored by risperidone, suggesting that these signaling pathways are possible targets of antipsychotic treatment and potential diagnosis biomarkers of schizophrenia.

MiR-25 was reported to be involved in epilepsy, Parkinson’s disease, cerebral ischemia, multiple sclerosis, panic disorder, alexithymia, and tic disorders (Guo et al., 2014; Zhang et al., 2016; Nuzziello et al., 2018; Min et al., 2019; Van der Auwera et al., 2022; Wang et al., 2022; Zhou and Qiao, 2022). The present study demonstrated that miR-25 might also be associated with schizophrenia, further confirming the role of miR-25 in neuropsychiatric diseases and suggesting that miR-25-3p might be a potential therapeutic target or a diagnosis biomarker of schizophrenia. It was that miR-25-3p is closely related with oxidative stress and apoptosis in neurons (Li et al., 2020). Extensive evidence indicates that oxidative stress and apoptosis is involved in the pathophysiology of schizophrenia (Patel et al., 2017). Therefore, the connection between oxidative stress and apoptosis and miR-25-3p in schizophrenia is worth a detailed investigation.

The current study also found increased SIK1 protein expression in the schizophrenic model rat brains, which is generally consistent with the findings of those previous studies on epilepsy and depression (Hansen et al., 2015; Pröschel et al., 2017; Liu et al., 2020). It should be noted that activation of the downstream CRTC2/CREB1 signaling requires the phosphorylation of SIK1 by LKB1 (Darling and Cohen, 2021). However, this study showed inconsistent activations of SIK1 and CRTC2 in both brain regions. We found up-regulated protein expression and phosphorylation levels of SIK1 in the CPu along with increased protein expression of LKB1, whereas the ratio of p-SIK1/SIK1 does not support a significant change in SIK1 phosphorylation in the schizophrenic model rats. Hollstein et al.’s (2019) study also showed elevated protein expression and phosphorylation levels of SIK1 as well as increased LKB1 levels in lung cancer cells, however, the ratio of p-SIK1/SIK1 was not shown in that study. Unfortunately, the reason for the inconsistent phosphorylation of SIK1 and CRTC2 presented in our study cannot be explained by the present data. Therefore, an in-depth study is required to further verify the involvement of the SIK1/CRTC2/CREB1 signaling pathway in schizophrenia.

Besides SIK1, TWIST1 is another direct downstream responder of miR-25-3p (Yu et al., 2021). TWIST1 expresses in embryonic and fetal human brain neurons (Elias et al., 2005) and elevated TWIST1 expression in various brain regions was reported to be associated with neuropsychiatric disorders (He et al., 2021). In the present study, we found that the TWIST1 protein levels were up-regulated by the repeated MK-801 administration, suggesting a possible connection with schizophrenia. This finding extended the understanding on the role of TWIST1 in neuropsychiatric disorders. Furthermore, the involvement of GSK3β in the pathophysiology of schizophrenia has been well-documented (Emamian, 2012). Additionally, it has been confirmed that GSK3β can be phosphorylated by TWIST1 through the activation of the PI3K/Akt signaling pathway (Li and Zhou, 2011). Our results showed generally similar phosphorylating statuses of PI3K, Akt, and GSK3β caused by the repeated MK-801 administration in the rat brains. These findings suggest that miR-25-3p-mediated TWIST1/Akt/PI3K/GSK3β signaling pathway is very likely to be associated with the pathophysiology of schizophrenia.

**FIGURE 7:**
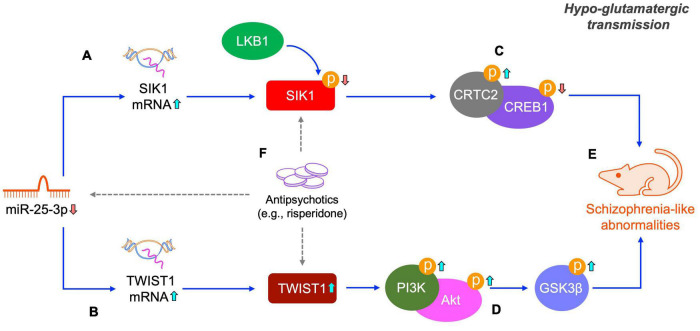
Schematic of the miR-25-mediated SIK1/CRTC2/CREB1 and TWIST1/PI3K/Akt/GSK3β signaling pathways in schizophrenia. Under a hypo-glutamatergic circumstance induced by MK-801, decreased miR-25-3p expression promotes the mRNA expression of SIK1 and TWIST1 **(A,B)**. Altered SIK1 activation probably caused by both miR-25-3p and LKB1 induces the inactivation of the CRTC1/CREB1 signaling cascade **(C)** and changed TWIST1 expression represses the PI3K/Akt/GSK3β signaling cascade **(D)**, both of which eventually lead to schizophrenia-like abnormalities **(E)**. These two signaling pathways altered by repeated MK-801 administration can be restored by antipsychotic drugs (e.g., risperidone), however, the underly pharmacological actions of risperidone requires further elucidation (indicated by dashed arrows) **(F)**.

Antipsychotic drugs are the primary treatments of schizophrenia. However, all current antipsychotic drugs have limited efficacy in treating schizophrenia in clinic. Therefore, searching new molecular targets and developing novel antipsychotic drugs is always the focus of scientific research in the field of schizophrenia. In the present study, we found that risperidone was able to restore the altered activities of the SIK1/CRTC2/CREB1 and TWIST1/PI3K/Akt/GSK3β signaling pathways induced by the repeated MK-801 administration (Figures 7A–E). It has been extensively reported that the CREB1 and Akt/GSK3β signaling can be regulated by various antipsychotic drugs (Emamian, 2012; Wang et al., 2018). It should be also noted that the CREB1 and Akt/GSK3β signaling is the downstream responders of the dopamine D_2_ receptor which is the main target of many antipsychotic drugs, including risperidone. Therefore, how risperidone exactly affected the CREB1 and Akt/GSK3β activities in this study remains to be elaborated, and in-depth studies are required to answer this question (Figure 7F).

The current study established a hypo-glutamatergic developmental schizophrenic rat model using repeated MK-801 administration. MK-801 is a non-competitive NMDAR antagonist. It was revealed that blockade of NMDA receptors alters the serotoninergic and dopaminergic systems in the brain (Meltzer et al., 2011; Kruse and Bustillo, 2022). In addition, risperidone possesses high binding affinity for the serotonin 5-HT_2_ and dopamine D_2_ receptor (Cohen, 1994) and was shown to be able to affect miR-25 expression in the present study. Therefore, it can be speculated that miR-25 and its downstream signaling pathways might be regulated by the serotoninergic and dopaminergic systems, in particular, the 5-HT_2_ and dopamine D_2_ receptor, which might also explain the association of miR-25 and other neuropsychiatric diseases that are related to the serotoninergic and dopaminergic systems (e.g., epilepsy and Parkinson’s disease). However, the exact underlying mechanism(s) require further explorations. Furthermore, it was previously reported that motor hyperactivity (resembling the positive symptoms of schizophrenia) was caused in animals by single/acute administration with non-competitive NMDAR antagonists [including MK-801, ketamine, and PCP (phencyclidine)]; in addition, repeated administration with non-competitive NMDAR antagonists induced impaired spatial learning and memory, cognition, and social interactions (resembling the negative symptoms of schizophrenia) (Winship et al., 2019). The present study employed sub-chronic repeated MK-801 administration only, thus the present model can only partly mimic schizophrenia and other administration regimens are required to consolidate the findings of this study. Moreover, schizophrenia manifests a number of clinical symptoms which cannot be completely mimicked in NMDA hypofunction schizophrenic models. Therefore, other schizophrenic models that possess different advantages in modeling schizophrenia [e.g., the gestational methylazoxymethanol (MAM) model, neonatal hippocampal lesion model, genetic animal models, patient-derived induced pluripotent stem cells (iPSCs), etc.] are also required to further validate the current findings.

In conclusion, the present study suggests that miR-25-3p-mediated SIK1/CRTC2/CREB1 and TWIST1/PI3K/Akt/GSK3β signaling pathways could be potential therapeutic targets for future antipsychotic drugs and candidate diagnosis biomarkers of schizophrenia.

## Data availability statement

The data presented in this study are deposited in the NCBI’s Gene Expression Omnibus (GEO) repository, accession number GSE220510.

## Ethics statement

The animal study was reviewed and approved by the Animal Ethics Committee of Yangzhou University Medical College (Ethics No.: YXYLL-2020-53).

## Author contributions

BP, XZ, and YL contributed to the conception and design of the study and wrote the first draft of the manuscript. BP, XZ, BH, JW, and YW performed the animal administration. XZ, BH, JW, and YW performed Western blots and qRT-PCR. BP, XZ, BH, and JW analyzed and interpreted the data. All authors contributed to manuscript revision, read, and approved the submitted version.
