# Supplementary material for: The SIK1/CRTC2/CREB1 and TWIST1/PI3K/Akt/GSK3β signaling pathways mediated by microRNA-25-3p are altered in the schizophrenic rat brain
Source: Front Cell Neurosci. 2023 Jan 20;17:1087335. doi: 10.3389/fncel.2023.1087335 (PMC9896578; doi:10.3389/fncel.2023.1087335)
Supplement: Supplementary file 1 [file Image_1.PDF]

Figure S1. Uncropped membrane of the bands presented in Figure 3.

Figure S1A

PFC

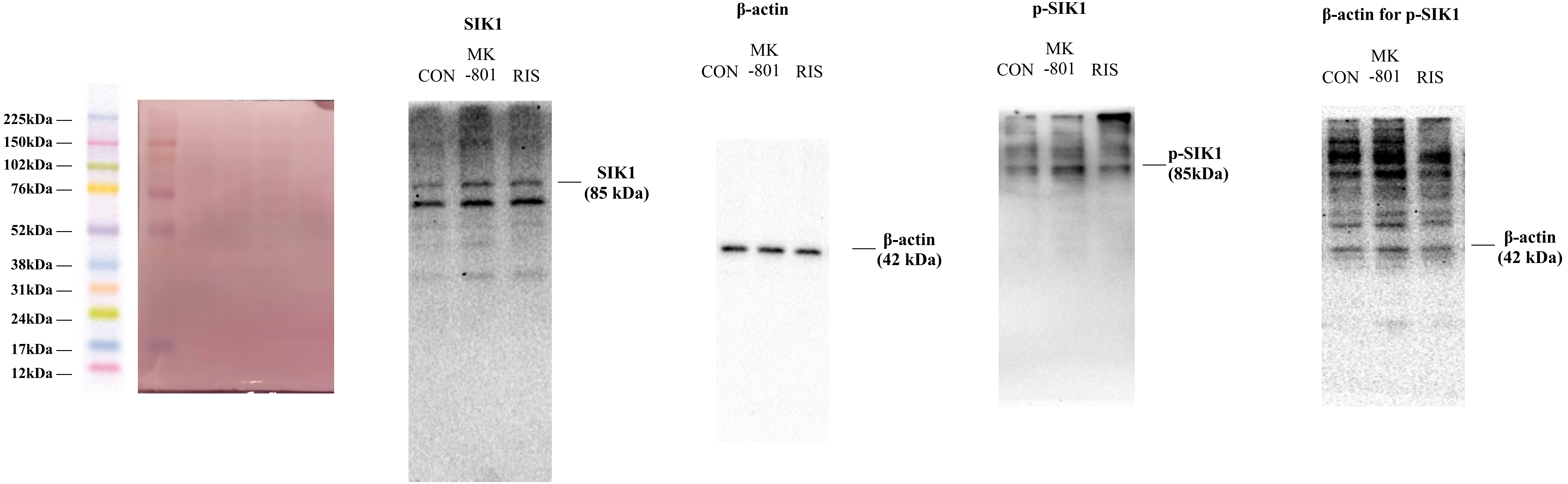

Figure S1B

PFC

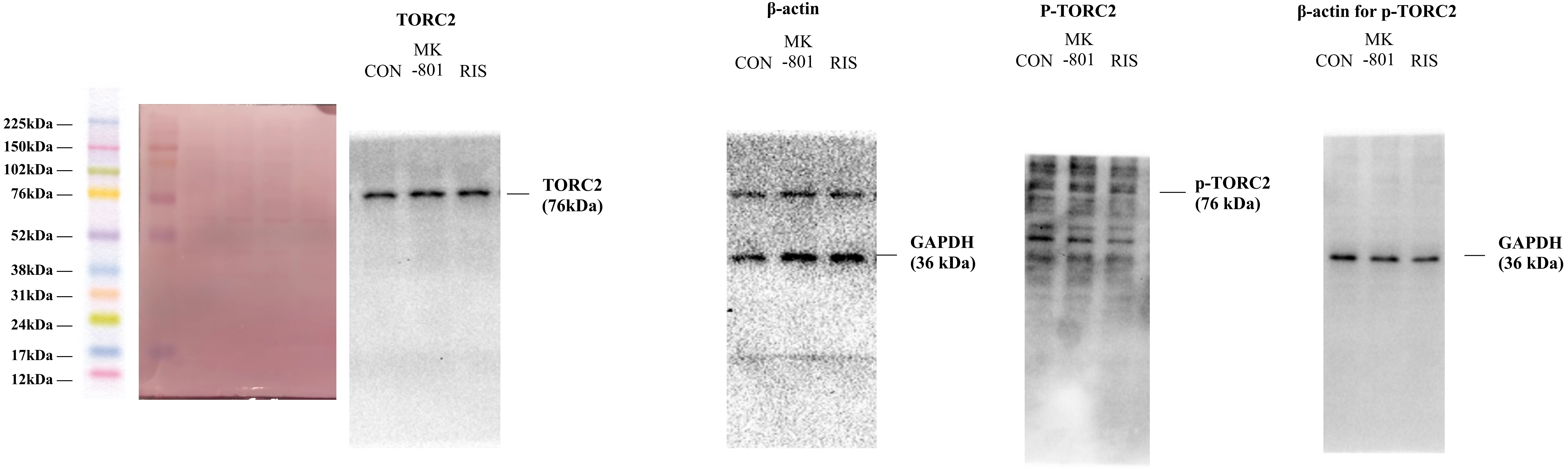

Figure S1C

PFC

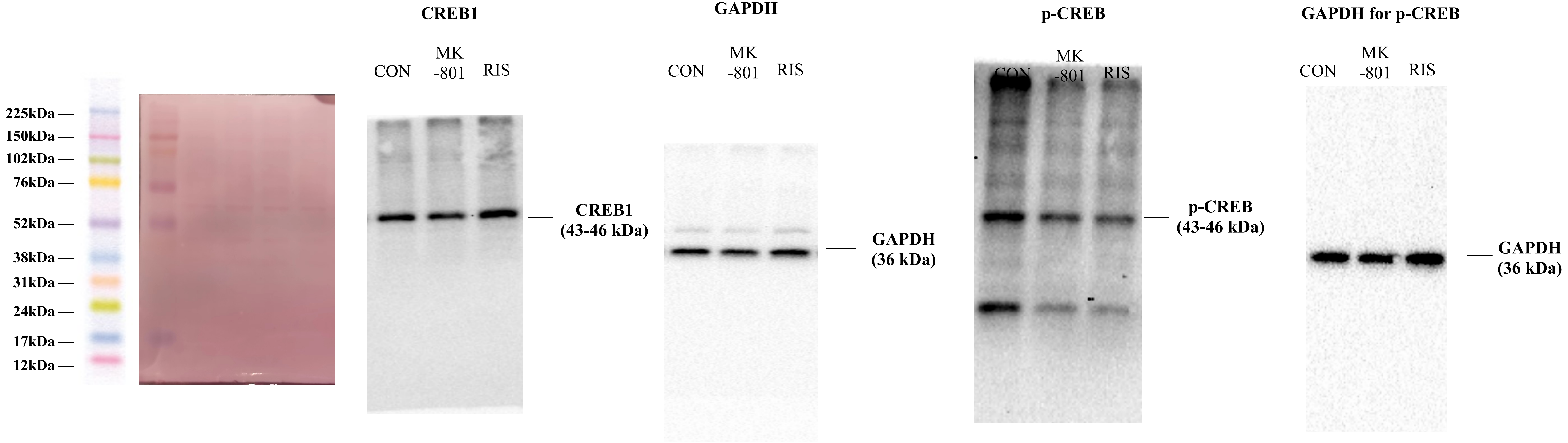

Figure S1D

PFC

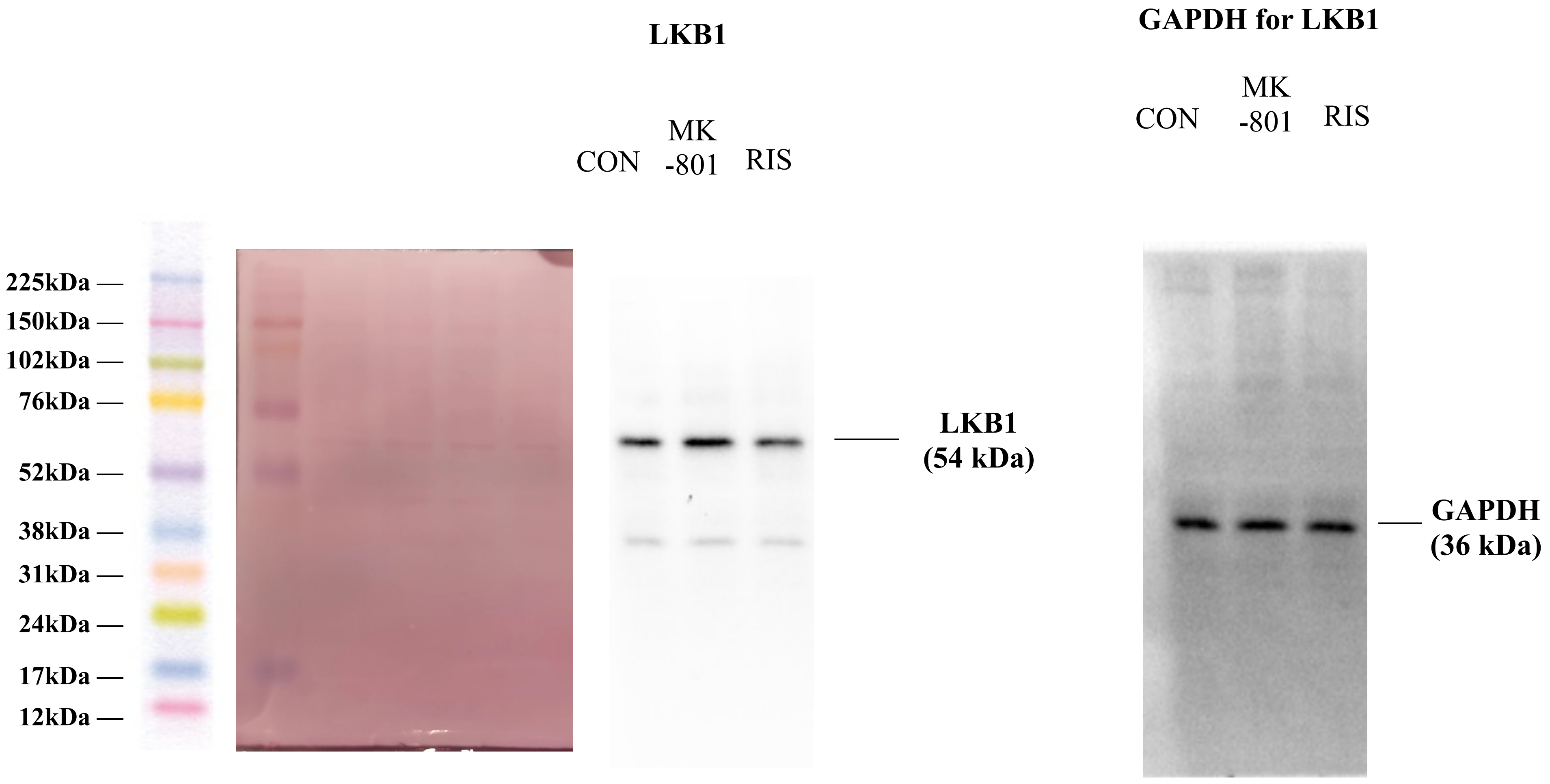

Figure S2. Uncropped membrane of the bands presented in Figure 4.

Figure S2A

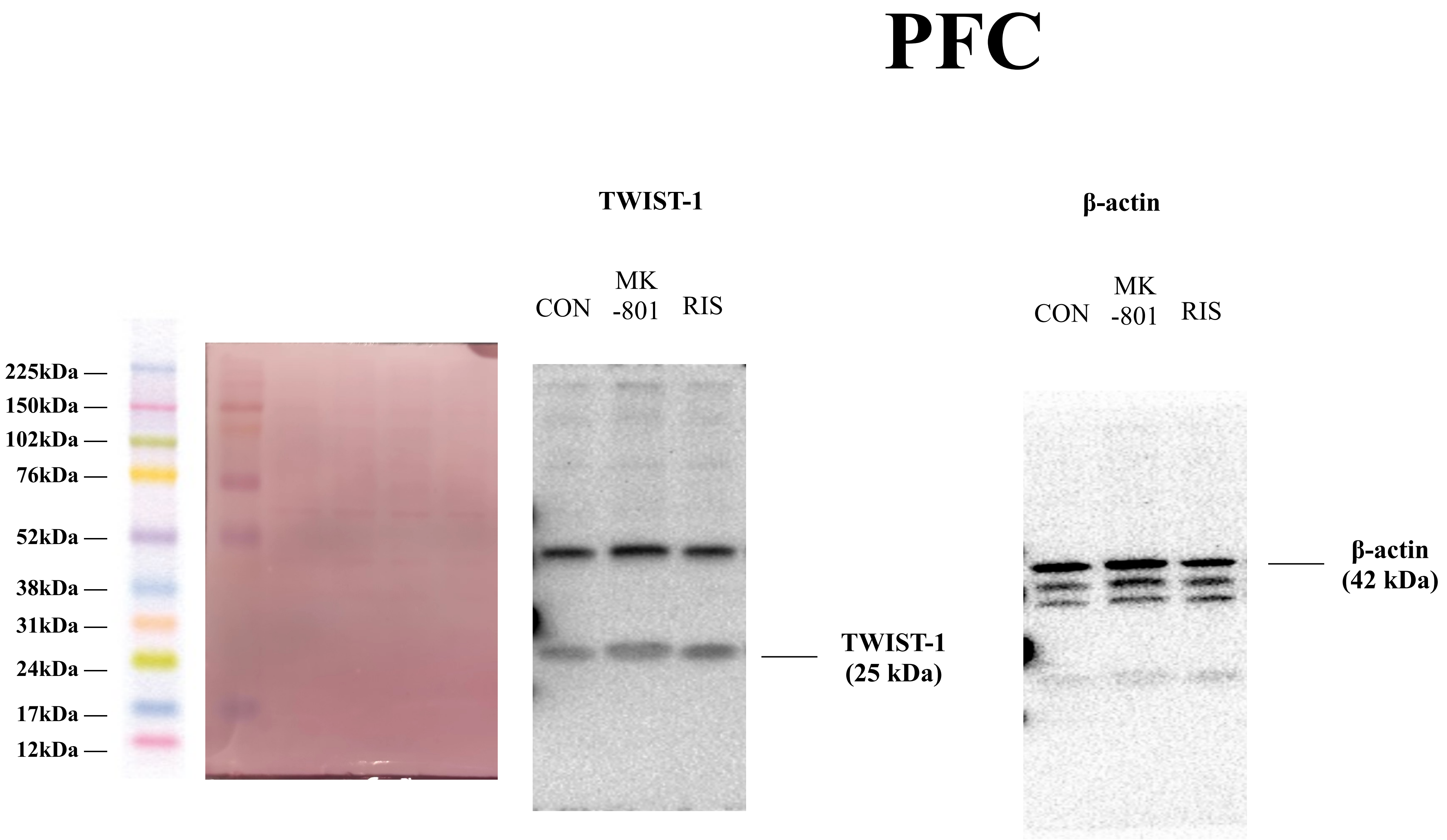
PFC
TWIST-1
β-actin
CON
MK -801
RIS
CON
MK -801
RIS
225kDa
150kDa
102kDa
76kDa
52kDa
38kDa
31kDa
24kDa
17kDa
12kDa
TWIST-1 (25 kDa)
β-actin (42 kDa)

Figure S2B

PFC

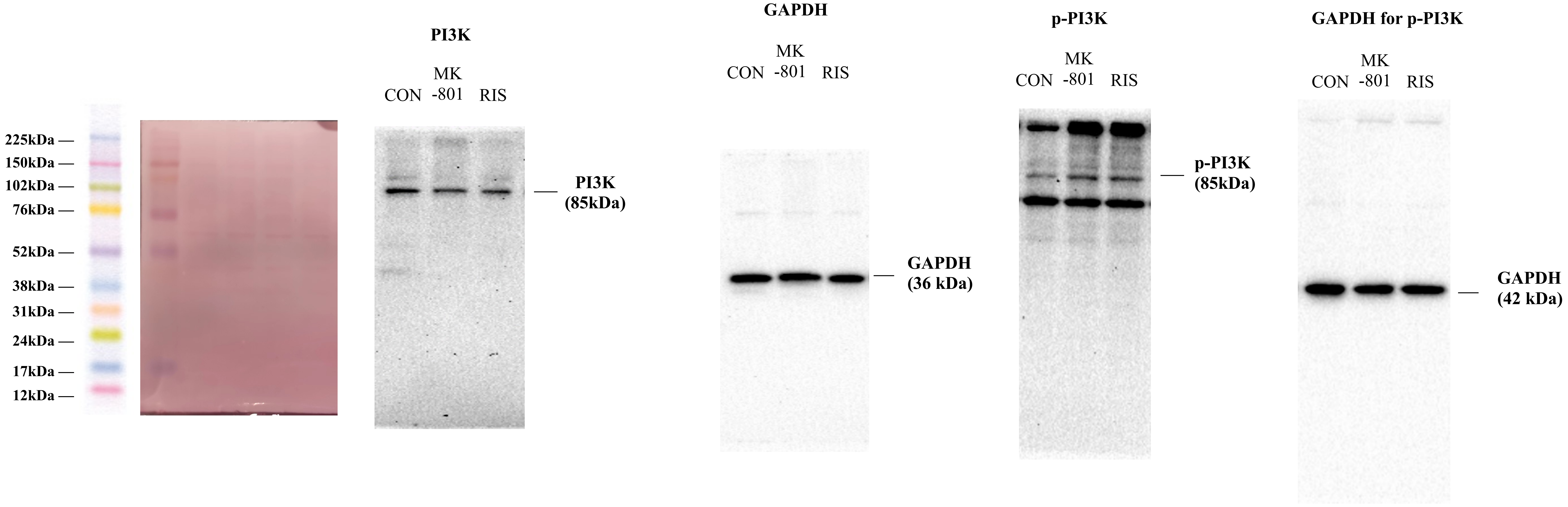

Figure S2C

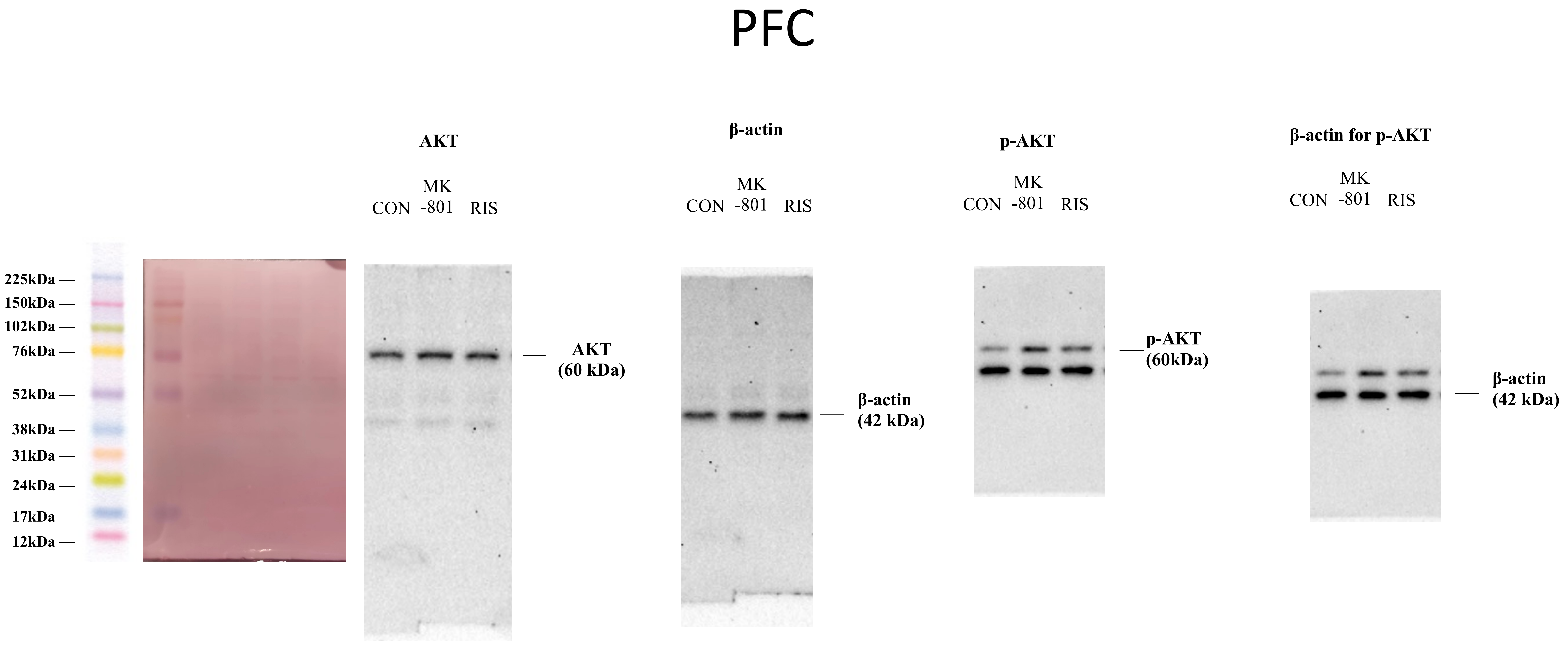
PFC
AKT
β-actin
p-AKT
β-actin for p-AKT
CON
MK -801
RIS
225kDa
150kDa
102kDa
76kDa
52kDa
38kDa
31kDa
24kDa
17kDa
12kDa
AKT (60 kDa)
β-actin (42 kDa)
p-AKT (60kDa)
β-actin (42 kDa)

Figure S2D

PFC

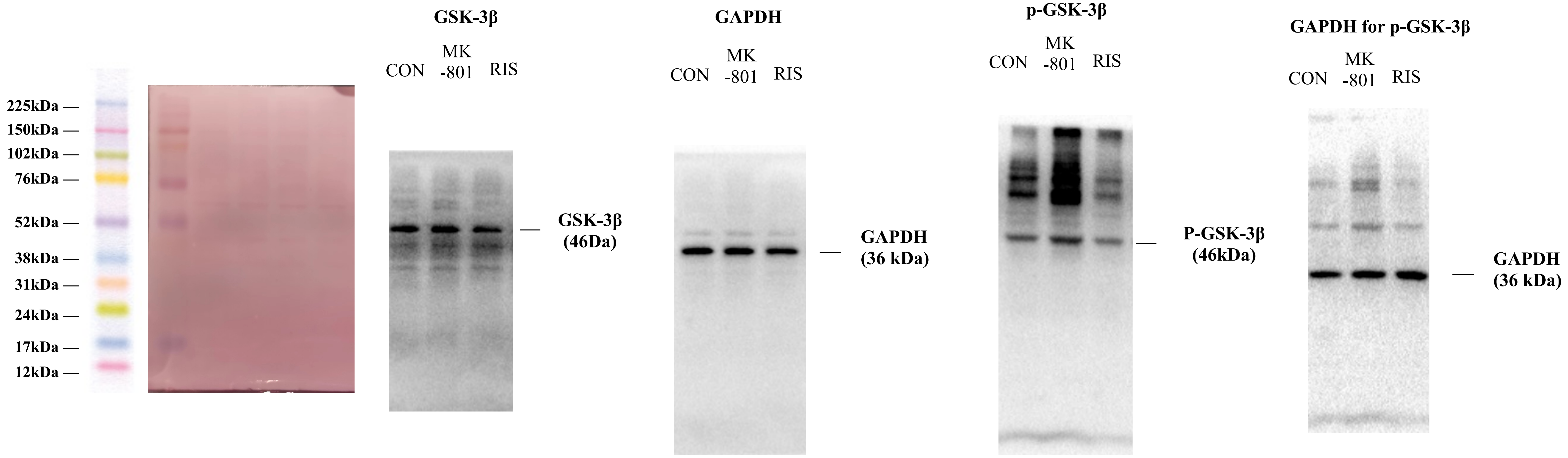

Figure S3. Uncropped membrane of the bands presented in Figure 5.

Figure S3A

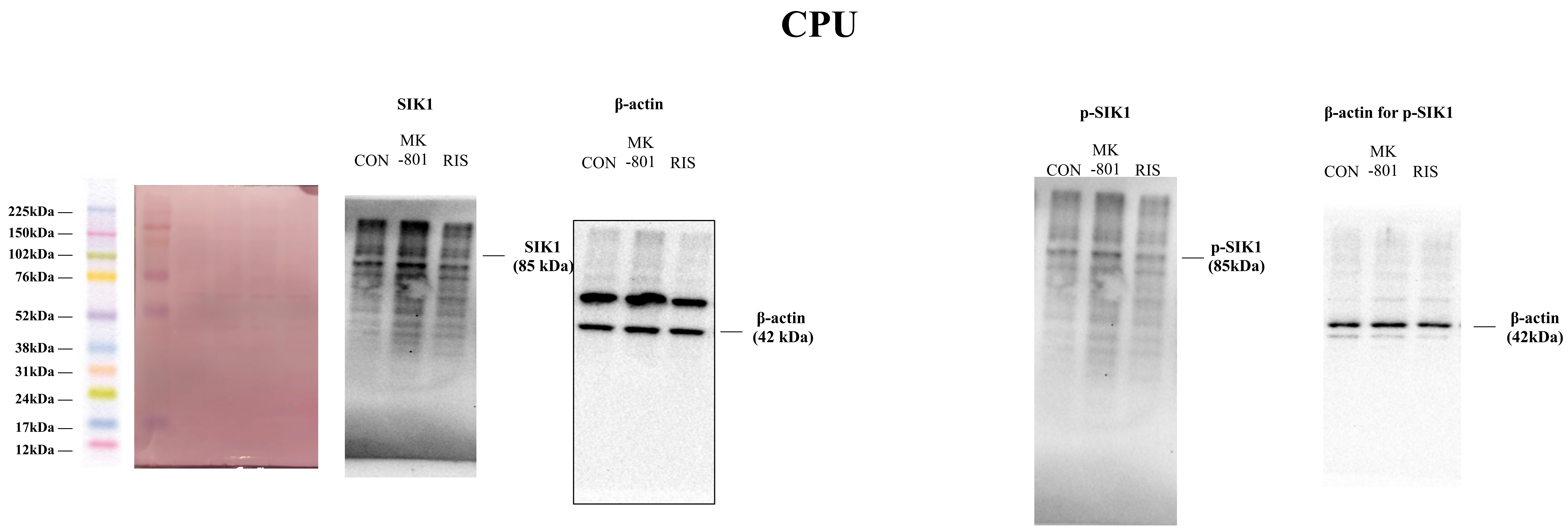
CPU
SIK1
β-actin
p-SIK1
β-actin for p-SIK1
CON
MK -801
RIS
225kDa
150kDa
102kDa
76kDa
52kDa
38kDa
31kDa
24kDa
17kDa
12kDa
SIK1 (85 kDa)
β-actin (42 kDa)
p-SIK1 (85kDa)
β-actin (42kDa)

Figure S3B

CPU

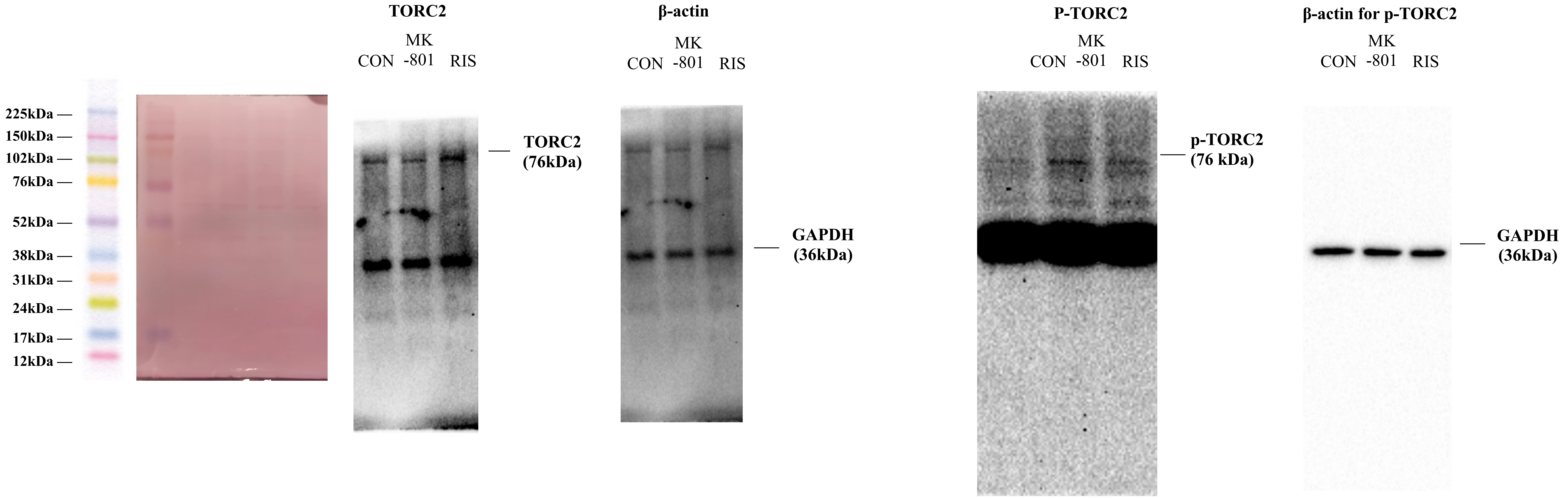

Figure S3C

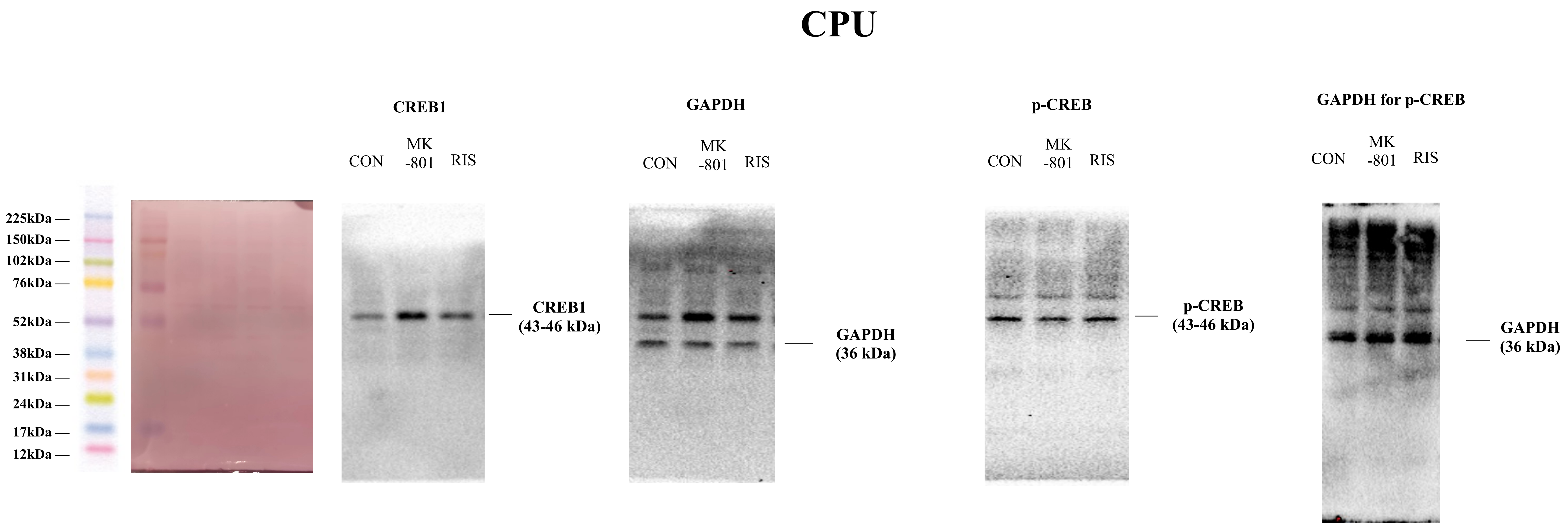
CPU
CREB1
GAPDH
p-CREB
GAPDH for p-CREB
CON
MK -801
RIS
225kDa
150kDa
102kDa
76kDa
52kDa
38kDa
31kDa
24kDa
17kDa
12kDa
CREB1 (43-46 kDa)
GAPDH (36 kDa)
p-CREB (43-46 kDa)
GAPDH (36 kDa)

Figure S3D

CPU

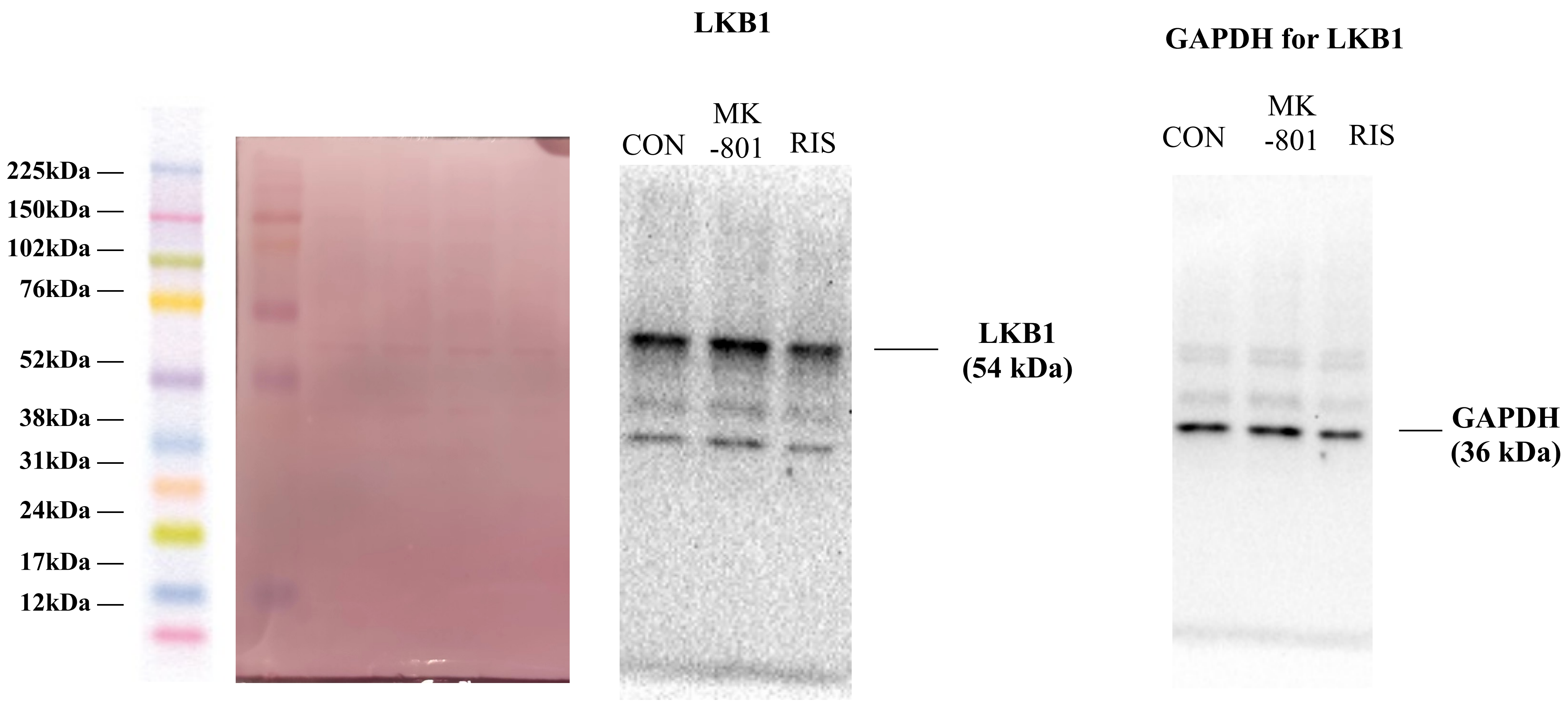

Figure S4. Uncropped membrane of the bands presented in Figure 6.

Figure S4A

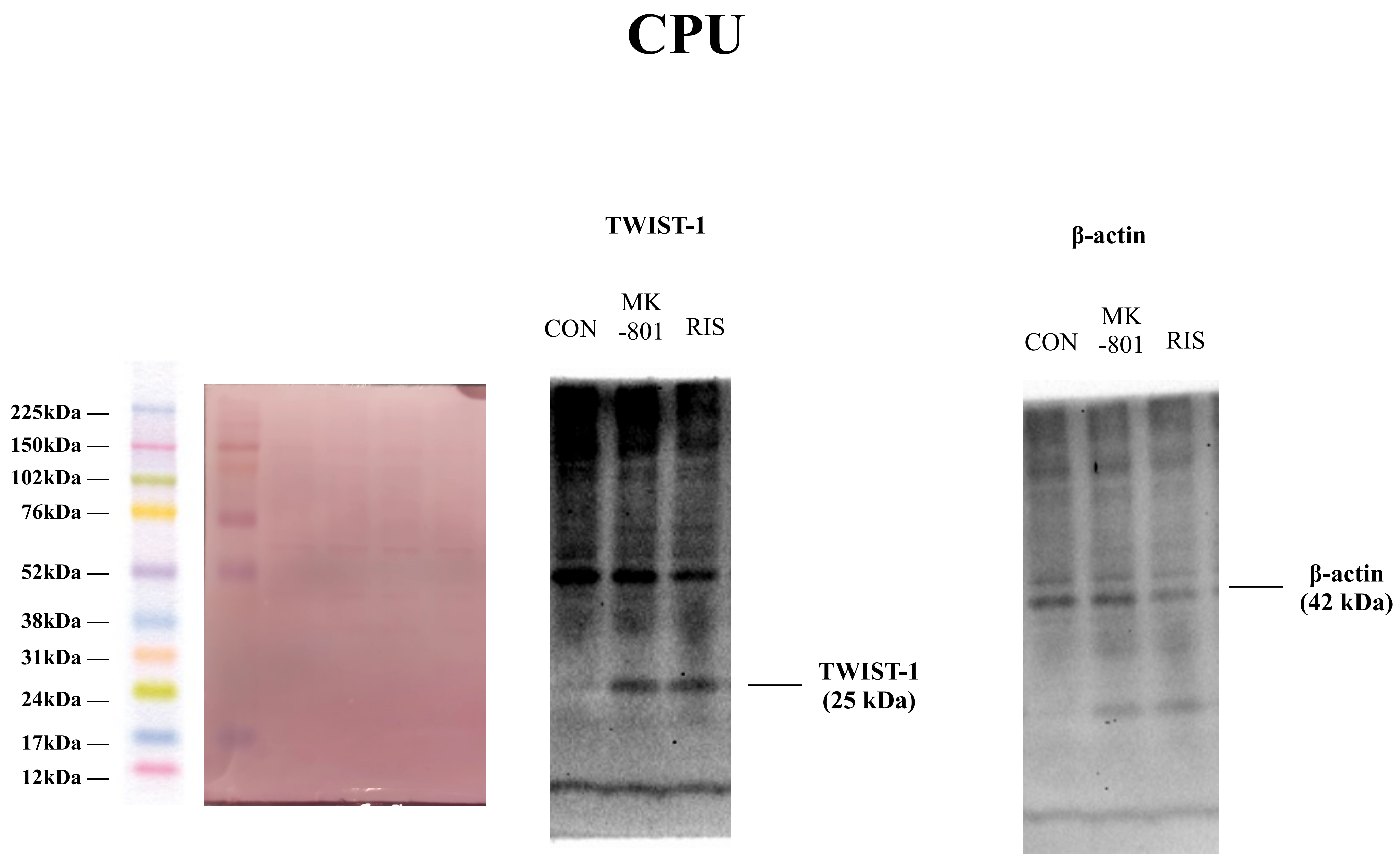
CPU
TWIST-1
β-actin
CON
MK
-801
RIS
CON
MK
-801
RIS
225kDa
150kDa
102kDa
76kDa
52kDa
38kDa
31kDa
24kDa
17kDa
12kDa
TWIST-1
(25 kDa)
β-actin
(42 kDa)

Figure S4B

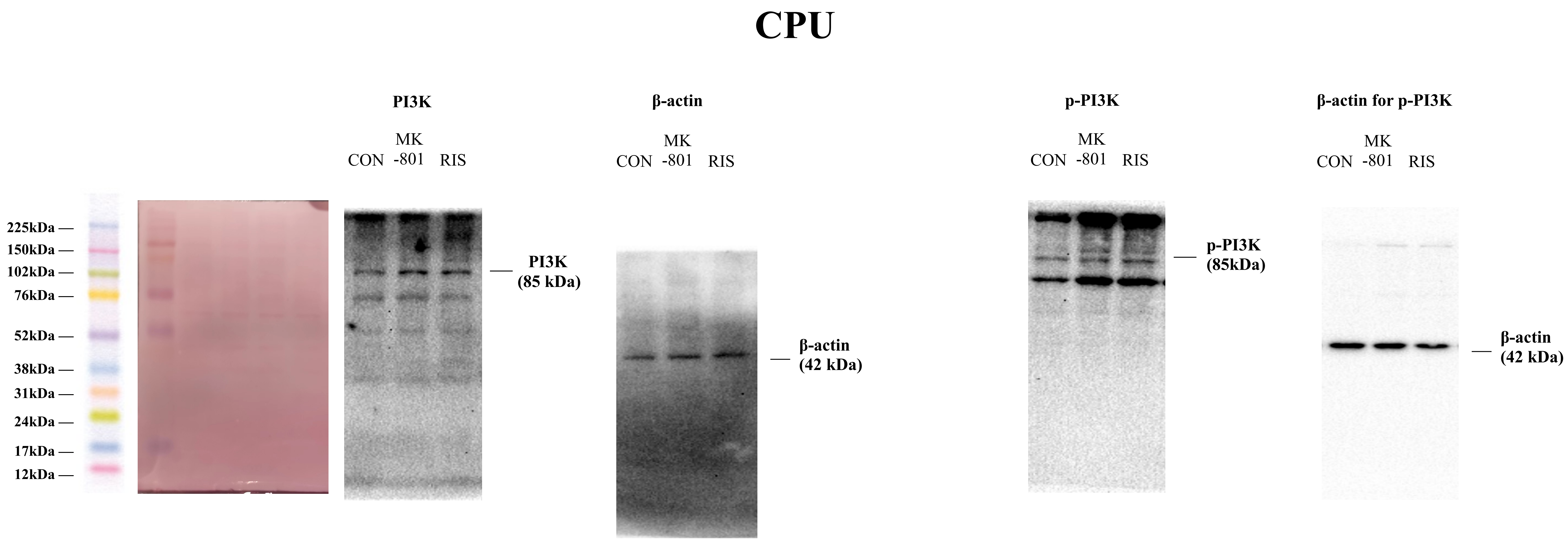
CPU
PI3K
β-actin
p-PI3K
β-actin for p-PI3K
MK
CON -801 RIS
MK
CON -801 RIS
MK
CON -801 RIS
MK
CON -801 RIS
225kDa
150kDa
102kDa
76kDa
52kDa
38kDa
31kDa
24kDa
17kDa
12kDa
PI3K
(85 kDa)
β-actin
(42 kDa)
p-PI3K
(85kDa)
β-actin
(42 kDa)

Figure S4C

CPU

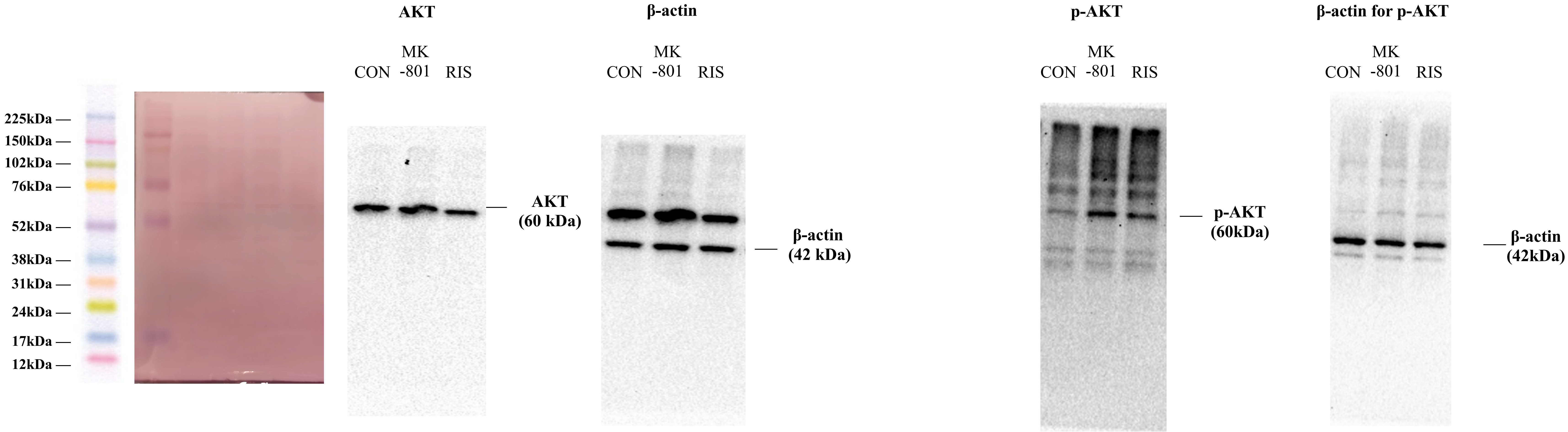

Figure S4D

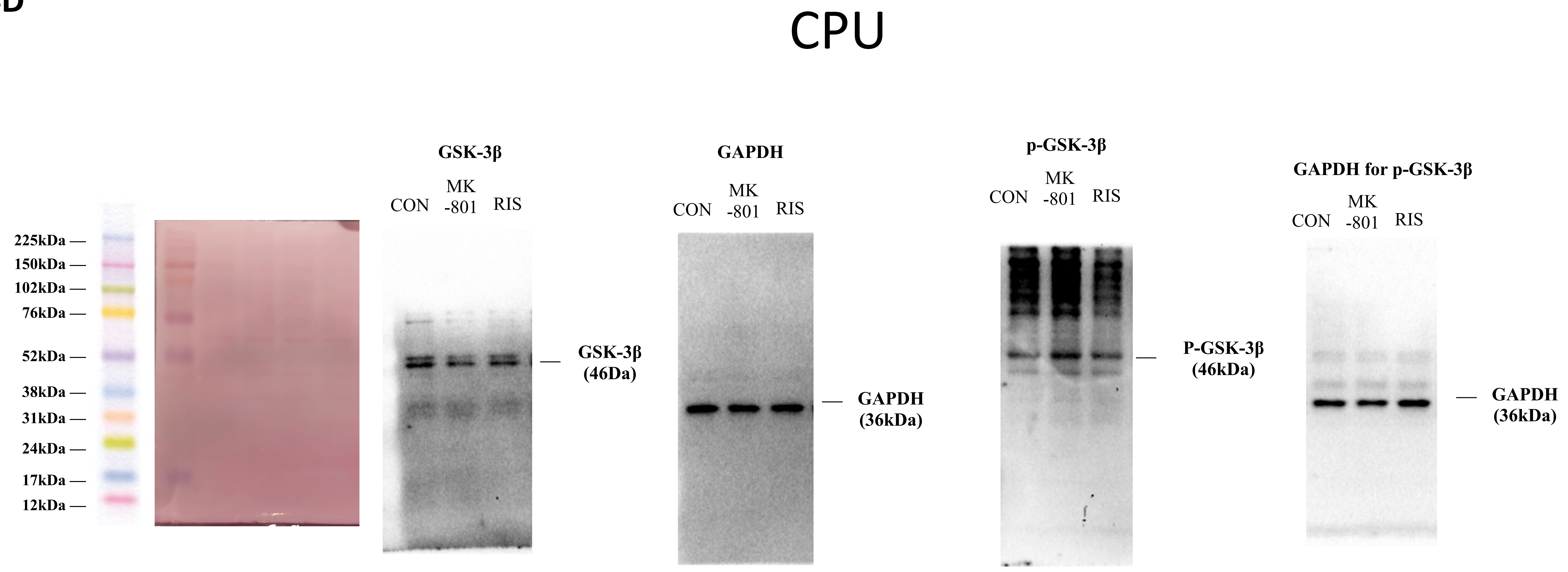
CPU
225kDa
150kDa
102kDa
76kDa
52kDa
38kDa
31kDa
24kDa
17kDa
12kDa
GSK-3β
CON
MK -801
RIS
GSK-3β (46Da)
GAPDH
CON
MK -801
RIS
GAPDH (36kDa)
p-GSK-3β
CON
MK -801
RIS
P-GSK-3β (46kDa)
GAPDH for p-GSK-3β
CON
MK -801
RIS
GAPDH (36kDa)
