# Supplementary material for: The SIK1/CRTC2/CREB1 and TWIST1/PI3K/Akt/GSK3β signaling pathways mediated by microRNA-25-3p are altered in the schizophrenic rat brain
Source: Front Cell Neurosci. 2023 Jan 20;17:1087335. doi: 10.3389/fncel.2023.1087335 (PMC9896578; doi:10.3389/fncel.2023.1087335)
Supplement: Supplementary file 2 [file Table_1.DOCX]

Table S1. GO analysis of the differentially-expressed genes revealed by RNA-Seq.

| **ID** | **GOterm** | **Subgroup** | **Enrichment score** | **GeneRatio** | **pvalue** |
| --- | --- | --- | --- | --- | --- |
| GO:1903429 | regulation of cell maturation | Biological process | 4.18 | 4/78 | 6.55054E-05 |
| GO:0048167 | regulation of synaptic plasticity | Biological process | 4.05 | 8/78 | 8.91857E-05 |
| GO:0060041 | retina development in camera-type eye | Biological process | 3.64 | 6/78 | 0.00022866 |
| GO:0060291 | long-term synaptic potentiation | Biological process | 3.63 | 6/78 | 0.000234799 |
| GO:0043010 | camera-type eye development | Biological process | 3.52 | 8/78 | 0.000304424 |
| GO:0035914 | skeletal muscle cell differentiation | Biological process | 3.44 | 4/78 | 0.000359869 |
| GO:0090596 | sensory organ morphogenesis | Biological process | 3.40 | 7/78 | 0.000396064 |
| GO:0007605 | sensory perception of sound | Biological process | 3.28 | 6/78 | 0.000522167 |
| GO:0001654 | eye development | Biological process | 3.19 | 8/78 | 0.000643534 |
| GO:0060395 | SMAD protein signal transduction | Biological process | 3.17 | 4/78 | 0.000668534 |
| GO:0032421 | stereocilium bundle | Cellular component | 2.51 | 3/77 | 0.003071363 |
| GO:0032426 | stereocilium tip | Cellular component | 2.47 | 2/77 | 0.003384584 |
| GO:0033017 | sarcoplasmic reticulum membrane | Cellular component | 2.16 | 2/77 | 0.006844022 |
| GO:0098862 | cluster of actin-based cell projections | Cellular component | 2.00 | 4/77 | 0.009906585 |
| GO:0032420 | stereocilium | Cellular component | 1.58 | 2/77 | 0.026512645 |
| GO:0005667 | transcription regulator complex | Cellular component | 1.54 | 5/77 | 0.028950922 |
| GO:0009897 | external side of plasma membrane | Cellular component | 1.53 | 5/77 | 0.029472437 |
| GO:0016529 | sarcoplasmic reticulum | Cellular component | 1.49 | 2/77 | 0.032397795 |
| GO:0016528 | sarcoplasm | Cellular component | 1.36 | 2/77 | 0.043541901 |
| GO:0032592 | integral component of mitochondrial membrane | Cellular component | 1.35 | 2/77 | 0.044526914 |
| GO:0001786 | phosphatidylserine binding | Molecular function | 2.64 | 3/71 | 0.002308067 |
| GO:0071837 | HMG box domain binding | Molecular function | 2.39 | 2/71 | 0.004100364 |
| GO:0005184 | neuropeptide hormone activity | Molecular function | 2.28 | 2/71 | 0.005224954 |
| GO:0016503 | pheromone receptor activity | Molecular function | 2.06 | 3/71 | 0.008654501 |
| GO:0072341 | modified amino acid binding | Molecular function | 2.01 | 3/71 | 0.009871958 |
| GO:0001102 | RNA polymerase II activating transcription factor binding | Molecular function | 1.78 | 2/71 | 0.016428388 |
| GO:0005179 | hormone activity | Molecular function | 1.77 | 3/71 | 0.017057885 |
| GO:0140297 | DNA-binding transcription factor binding | Molecular function | 1.71 | 5/71 | 0.019687723 |
| GO:0004879 | nuclear receptor activity | Molecular function | 1.69 | 2/71 | 0.020609504 |
| GO:0098531 | ligand-activated transcription factor activity | Molecular function | 1.69 | 2/71 | 0.020609504 |
